# CPP2Vec: A representation learning approach for cell-penetrating peptides prediction

**DOI:** 10.1371/journal.pcbi.1014118

**Published:** 2026-07-13

**Authors:** Stavroula Svolou, Vasileios Konstantakos, Anastasia Krithara, Georgios Paliouras

**Affiliations:** 1 Institute of Informatics and Telecommunications, NCSR “Demokritos”, Agia Paraskevi, Greece; 2 Laboratory of Computational Biology, VIB Center for AI & Computational Biology (VIB.AI), Leuven, Belgium; 3 VIB-KU Leuven Center for Brain & Disease Research, Leuven, Belgium; 4 Department of Human Genetics, KU Leuven, Leuven, Belgium; Tel Aviv University, ISRAEL

## Abstract

**Background:**

Cell-penetrating peptides (CPPs) facilitate the delivery of a variety of therapeutic molecules across the plasma membrane, from small chemical substances to nucleic acid-based macromolecules, such as antisense oligonucleotides (ASOs). Among neutral ASOs, peptide nucleic acids (PNAs) and phosphorodiamidate morpholino oligomers (PMOs) have been extensively studied as potential medical treatments for Duchenne Muscular Dystrophy (DMD), a severe genetic disease that causes muscle degeneration progressively. Over the last few decades, many *in silico* methods have emerged to detect novel CPPs, counterbalancing the cost of wet-lab experiments.

**Results:**

In this study, we propose CPP2Vec, a Word2Vec-based CPP prediction method, where the Word2Vec technique is used to represent amino acid sequences of peptides. To address the limited sequence diversity, sparse biological grounding, and the still poorly understood mechanisms underlying CPPs uptake, we constructed CPP2Vec-GenSet, a hybrid dataset that integrates computationally generated peptides with experimentally curated CPPs. This combined resource provides a robust training foundation that supports reliable representation learning and enhances cross-task model performance. Using this framework, we developed three task-specific supervised machine learning models for CPP-Classification, Uptake-Efficiency and PMO-Delivery. The first two models were designed to determine if an unseen peptide is a CPP and to predict its uptake efficiency, respectively, while the PMO-Delivery model predicts whether a peptide could enhance the cellular delivery of a PMO-complex compared to its naked version. Furthermore, we explored an alternative approach using pre-trained protein-based Large Language Models (LLMs) – ProtT5, ProtBERT, and ESM-2 – to generate the embeddings, resulting in three task-specific models, namely CPP2LLM. Benchmarking against state-of-the-art CPP prediction tools demonstrates that CPP2Vec achieves robust predictive performance and generalization across tasks, while maintaining exceptional computational efficiency.

**Conclusion:**

In this research, we present a Machine Learning (ML)-based tool that introduces the use of the Word2Vec technique in the field of CPPs prediction. Notably, CPP2Vec automatically learns informative peptide representations directly from sequence data, generalizes reliably across multiple tasks, and achieves high predictive performance with minimal computational resources, providing a reproducible and practical *in silico* tool to support the early-stage identification and prioritization of CPPs with potential therapeutic relevance. CPP2Vec is available for use at: https://github.com/SSvolou/CPP2Vec.

## Author summary

Cells are surrounded by a membrane that functions as a highly selective security system, guarding against harmful invaders while maintaining internal stability. However, this same protective barrier also blocks many beneficial therapeutic molecules from reaching their intracellular targets, so even promising drugs may fail simply because they cannot cross it. Cell-penetrating peptides (CPPs) are short amino acid sequences capable of transporting molecular cargo across membranes. Acting as biological delivery vehicles, they can carry therapeutic compounds, including genetic medicines such as antisense oligonucleotides, directly into cells, opening new avenues for therapeutic applications, including the treatment of genetic disorders such as Duchenne Muscular Dystrophy. Despite their considerable potential, identifying CPPs and their uptake efficiency consistently remains a costly and time-intensive process when relying only on lab experiments. To address this challenge, we developed CPP2Vec, a computational tool designed to identify promising CPPs. CPP2Vec learns from known peptide sequences by detecting patterns in their amino acid composition and converting them into numerical representations that capture biologically meaningful features. To enhance learning, we constructed a hybrid dataset combining experimentally validated peptides with computationally generated sequences, improving diversity and generalization for CPP prediction. CPP2Vec aims to accelerate the identification of promising peptide-based therapies.

## 1 Introduction

As the therapeutic landscape expands at an increasing rate, the need to design drug delivery methods to improve therapeutic efficacy, minimize toxicity side effects, and enable innovative medical treatments emerges.

To this end, CPPs have been introduced to facilitate the delivery of a variety of therapeutic molecules across the plasma membrane, from small chemical substances to nucleic acid-based macromolecules. CPPs are short peptides comprised of 5–30 amino acids that are categorized based on their origin, conformation, and physical-chemical character into subgroups [1]. Even though the evolution of next-generation sequencing technologies accelerates peptide sequencing, the cost of wet-lab experimental studies remains high in terms of both time and resources.

For this reason, in the last few decades, many *in silico* – mainly ML-based – methods have been developed to detect novel CPPs, counterbalancing the cost of conventional in vitro assays [2]. Some of them, not only predict if a peptide is a CPP or not, but also categorize them depending on their predicted uptake efficiency into “high” or “low” classes. A crucial component in the development of prediction models is the technique that has been used for the sequence representation. Currently available tools use some conventional feature descriptors aiming to capture compositional or physicochemical properties of CPPs, such as amino acid composition (AAC), composition of K-spaced amino acid pairs (CKS) and pseudo amino acid composition (PseAAC) [3–6], while others are based on binary encoding [7].

A summary of existing ML-based CPP prediction tools is provided in Table 1. By organizing and benchmarking CPP2Vec alongside a wide range of existing CPP predictors, including classical machine learning, ensemble, and deep learning approaches, our study provides a clear overview of methodological diversity and ensures that CPP2Vec is positioned within the full spectrum of current CPP-specific computational strategies. Random Forest remains the most frequently adopted classification algorithm, though various studies have explored alternatives such as Support Vector Machines, ensemble strategies, and deep learning architectures. Among these tools, only a subset – namely CPPred-RF, MLCPP, Wolfe et al., DeepCPPred, and MLCPP 2.0 – explicitly support both CPP classification and uptake efficiency prediction. To the best of our knowledge, Wolfe et al. is currently the only method specifically designed to predict the functional delivery efficacy of CPP-PMO conjugates, as assessed by activity-based thresholds in antisense performance. Here, “delivery efficacy” refers to functional antisense activity measured experimentally, which integrates multiple processes such as cellular uptake, endosomal escape, and nuclear delivery, and thus serves as practical proxy for overall PMO delivery performance.

**Table 1:** Brief overview of existing state-of-the-art ML-based prediction tools.

Year	Name	Method	CPP Classification	Uptake Efficiency	PMO Delivery
2013	CellPPD [7]	SVM	✓	✕	✕
2016	Diener et al. [8]	RF - SVM	✓	✕	✕
2016	C2Pred [9]	SVM (Feature Selection)	✓	✕	✕
2017	SkipCPP-Pred [4]	RF	✓	✕	✕
2017	CPPred-RF [5]	RF	✓	✓	✕
2018	MLCPP [10]	Extra Trees - RF	✓	✓	✕
2018	KELM-CPPpred [3]	Kernel-based Extreme ML	✓	✕	✕
2018	BChemRF-CPPred [11]	RF	✓	✕	✕
2018	CPPred-FL [6]	RF	✓	✕	✕
2018	Wolfe et al. [12]	RF	✓	✓	✓
2021	DeepCPPred [13]	CNN (Deep Learning)	✓	✓	✕
2022	MLCPP 2.0 [14]	Stacked Ensemble Learning	✓	✓	✕
2025	pLM4CPPs [15]	CNN	✓	✕	✕
2026	**CPP2Vec**	RF - SVM	✓	✓	✓

SVM: Support Vector Machine. RF: Random Forest Classifier. CNN: Convolutional Neural Network. The tool described in this work is marked in bold.

It should be noted that some of these published tools were not included in our benchmarking due to inaccessible web servers, lack of publicly available source code, or persistent runtime errors during testing. While these methods remain of interest, incorporating them posed significant reproducibility challenges and fell outside the scope of this study, which prioritizes methodological relevance, consistency, and the use of accessible and operational tools.

In this study, we explored the usage of LLMs to produce contextualized embeddings for amino acid sequences in the field of CPP prediction. We evaluate the performance of protein-specific variants of pre-trained LLMs, namely ProtT5 [16], ProtBERT [17] and ESM-2 [18], while simultaneously we introduce CPP2Vec, a Word2Vec-based CPP prediction method, where the Word2Vec (W2V) [19] technique is used to generate the representations of amino acid sequences of peptides. Our proposed method not only classifies uncharacterized peptides into CPP/non-CPP and high/low uptake efficiency categories, but also provides a reliable model for predicting whether a peptide can enhance the delivery of a PMO-complex into the cell compared to the unconjugated PMO. Finally, we include a case study for DMD [20,21] highlighting CPP2Vec’s potential usage in real-life scenarios.

## 2 Materials and methods

In this section, we summarize the approach that we followed to construct our proposed prediction models. Specifically, we describe our 4-stage CPP prediction pipeline, providing details about the selected training and test datasets, as well as information about how we evaluated the performance of our method.

### 2.1 Description of CPP prediction pipeline

**Fig 1:**
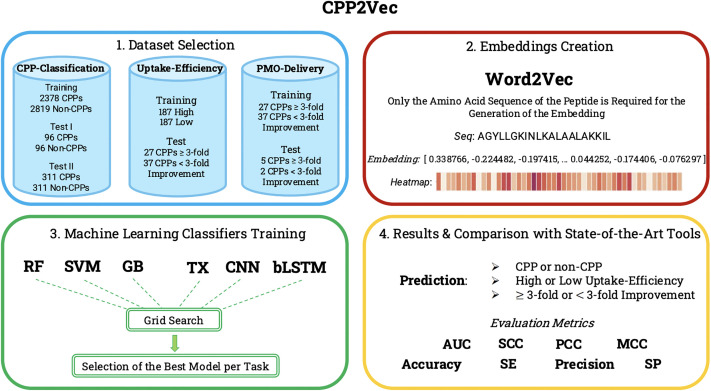
Workflow of CPP2Vec construction. (1) Dataset selection and data preprocessing. (2) Generation of contextualized embeddings through dataset-specific W2V approach. (3) Testing various ML algorithms to detect the proper one for each task. (4) Evaluation of the proposed models and comparison with state-of-the-art tools.

The workflow we implemented for CPP prediction is illustrated in Fig 1 and includes four main stages. The first stage involves dataset selection and preparation. To construct a high-quality prediction model, reliable and rigorous training and test datasets were needed to ensure unbiased and accurate results. The second stage is the generation of contextualized embeddings for CPP candidates. In this study, we utilized two approaches to represent amino acid sequences in a vectorized format. First, we employed a dataset-specific W2V technique, and afterward, we utilized the pre-trained protein LLMs: ProtT5 [16], ProtBERT [17], and ESM-2 [18]. The third stage is ML model construction. Various ML algorithms, including Support Vector Machine (SVM), Random Forest (RF), and Gradient Boosting (GB) classifiers, were tested to detect the best model for each task. Finally, the fourth stage involves the evaluation of the proposed models to confirm that the predicted results are trustworthy. A comparison of our approach with state-of-the-art tools was also conducted during this stage.

### 2.2 Preparation of datasets

Considering each of the tasks that we studied (i.e., CPP-Classification, Uptake-Efficiency and PMO-Delivery), we carefully selected training and test datasets. For the sake of consistency, we needed to make an assumption to ensure fair comparisons with state-of-the-art tools. Since none of these tools accept non-natural amino acids in the provided sequences, we substituted β-alanine with α-alanine and 6-aminohexanoic acid with lysine [22] and ambiguous amino acids were standardized by replacing leucine (L) for J, cysteine (C) for U, lysine (K) for O, and glutamic acid (E) for Z in all datasets [23].

#### 2.2.1 Construction of training datasets.

**Fig 2:**
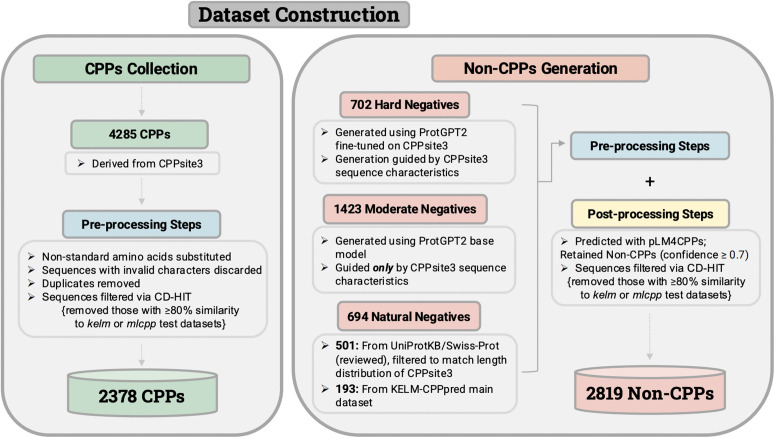
Overview of dataset construction for the CPP-Classification task. Positive samples (2,378 CPPs) were collected from CPPsite3 after preprocessing (non-standard amino acids replaced, invalid or duplicate sequences removed). Three categories of Non-CPPs were generated: 702 Hard Negatives using ProtGPT2 fine-tuned on CPPsite3, 1,423 Moderate Negatives using the base ProtGPT2 model, and 694 collected Natural Negatives, including 501 derived from UniProtKB/Swiss-Prot (reviewed) and 193 from the KELM-CPPpred main dataset. All Negative sequences were evaluated using pLM4CPPs (ESM-640), retaining only those predicted as Non-CPPs with confidence ≥ 0.7. CD-HIT clustering (80% similarity threshold) was applied to remove sequences overlapping with independent test datasets (*kelm* and *mlcpp*), and duplicates were discarded.

For the CPP-Classification task, we constructed a novel hybrid dataset, summarized in Fig 2. Positive samples (2,378 CPPs) were collected from CPPsite3 [24] after preprocessing, including replacement of non-standard/ambiguous amino acids, removal of invalid sequences, and elimination of duplicates.

Negative samples were generated from three sources: hard negatives (702 sequences) using ProtGPT2 [25] fine-tuned on CPPsite3, moderate negatives (1,423 sequences) using the base ProtGPT2 model, and natural negatives (501 sequences) derived from UniProtKB/Swiss-Prot [26] (reviewed), a high quality of manually annotated and non-redundant protein sequences database. Moderate negatives rely solely on statistics from the CPPsite3 dataset (average length, minimum/maximum lengths, and most common starting amino acids) to guide sequence generation. The resulting sequences are CPP-like in general length and starting amino acid composition, but they are not fine-tuned to mimic CPP-specific patterns, as with hard negatives. Natural negatives were sampled to match the length distribution of CPPsite3 sequences, preserving the average length (∼18 amino acids) of the positive dataset. All generated negatives were evaluated using pLM4CPPs (ESM-640) [15], retaining only sequences predicted as Non-CPPs with confidence ≥ 0.7. CD-HIT [27] clustering (80% similarity threshold) was applied to remove sequences overlapping with independent *kelm* and *mlcpp* test datasets, to ensure fair and comprehensive evaluations. Additionally, a subset of negative peptides from the original KELM-CPPpred main dataset [3] was included. These were originally assembled from CAMP_R3_ [28] and BIOPEP [29] sequences: 1,224 CAMP_R3_ peptides reduced to 704 non-redundant sequences, 1,426 BIOPEP peptides filtered to 326, followed by random selection and inclusion of 34 experimentally validated non-CPPs from Sanders et al. [30], yielding 408 sequences. After applying our filtering and redundancy reduction criteria, only 194 out of 408 sequences were retained in the final training dataset. In total, our generated training dataset-hereafter referred to as CPP2Vec-GenSet-comprises 2,378 CPPs and 2,819 negative sequences.

For the Uptake-Efficiency task, we utilized for training the CPPsite3_Gautam dataset, proposed by Gautam et al. [7] in 2013. This dataset is derived from the CPPsite database [31], the predecessor of the CPPsite 2.0 database, which contains 843 experimentally validated CPPs. Among these, 430 peptides were collected from research publications, 172 from patents and 241 from both sources. The majority of the peptides (522) are protein derived, while the rest are either synthetic (278) or chimeric (43). To maintain consistency and biological relevance, peptides containing non-natural amino acids or D-amino acids were excluded-since such components are rarely involved in typical biological systems-resulting in a refined dataset of 708 unique, naturally composed peptides. This filtering step was intended to ensure uniformity and improve the applicability of predictions in natural contexts. These CPPs were tested for their cell penetration capability across various cell types and experimental conditions. According to the original CPPsite database classifications (later adopted in the CPPsite3_Gautam dataset), uptake efficiency was categorized as low (<25%), medium (26–75%), or high (>75%) based on the percentage of cellular internalization relative to a control peptide [14]. Peptides labeled as “high” uptake in Gautam et al.’s dataset therefore correspond to this high-efficiency category (>75%). Since experimentally validated Non-CPPs were scarce, the high-uptake CPPs were paired with randomly selected peptides from SwissProt [32] to form the CPPsite3_Gautam dataset, comprising 187 high-efficiency CPPs and 187 low-efficiency peptides (374 total).

For the PMO-Delivery task, our proposed model aims to determine whether conjugating a given peptide would enhance PMO activity by at least 3-fold. In order to accomplish this, we selected as our training set the 64-peptides dataset generated by Wolfe et al. [12] For our classification purposes, we assigned label 1 to peptides that demonstrated a 3-fold or greater improvement in eGFP fluorescence compared to unconjugated PMOs, while assigning label 0 to others, a concept initially described in the same study.

#### 2.2.2 Independent test datasets.

For the CPP-Classification task, we validated the performance of our proposed model using two independent datasets: KELM-CPPpred [3] Independent and MLCPP [10] Independent datasets. Both datasets were adopted exactly as provided by the original authors, without any modification, reconstruction or additional preprocessing. For the sake of simplicity, they will be denoted as *kelm* and *mlcpp*, respectively.

The *kelm* dataset comprises 99 CPPs and 99 Non-CPPs originally compiled by Gautam et al. [7], and later adapted by the KELM-CPPpred study [3]. The CPPs were manually curated from recent research papers and patents reporting experimentally validated cell-penetrating peptides, ensuring that these sequences had not been used during training, feature selection, or parameter optimization. The Non-CPPs were selected from the SwissProt database [32], and were assumed not to possess cell-penetrating ability. To avoid redundancy, any sequences showing greater than >80% similarity to each other or to the training dataset were removed. After filtering, the final dataset consists of 96 CPPs and an equal number of Non-CPPs.

Concerning the *mlcpp* dataset, positive samples were extracted from CPPsite 2.0 [33], while negative samples were generated from SwissProt [32]. During the production of negative samples, peptides exhibiting sequence similarity to known CPPs were excluded to avoid potential false negatives. Following this step, the CD-HIT [27] program was applied using a sequence identity cutoff of 0.8 to remove redundant sequences. In addition, sequences overlapping with the authors’ benchmarking dataset or with the training datasets of previously published predictors (i.e., C2pred [9], CellPPD [7], and CPPpred [34]) were removed. The final dataset consists of 311 CPPs and 311 Non-CPPs (622 peptides in total).

For the Uptake-Efficiency task, we evaluated our proposed model using the 64-peptides dataset reported by Wolfe et al. [12]. The dataset quantifies functional PMO activity, which could be treated as a practical proxy for overall delivery performance, encompassing uptake, endosomal escape, and nuclear delivery. Following the activity criterion established in the original study, peptides producing a ≥ 3-fold increase in eGFP fluorescence relative to unconjugated PMO were assigned to the high uptake efficiency class (i.e., 27 peptides), while the remaining peptides were assigned to the low uptake efficiency class (i.e., 37 peptides).

Finally, for the PMO-Delivery task, we utilized as a test set the 7 peptides proposed by Wolfe et al. [12] in the same study. In that work, the authors synthesized PMO-CPP conjugates and evaluated them using a fluorescence reporter assay in HeLa-654 cells. These cells contain an engineered exon-skipping system: when the PMO enhances correct splicing of a mutant β‑globin intron, functional eGFP is expressed, and fluorescence is measured via flow cytometry – meaning that increased GFP corresponds directly to enhanced PMO internalization and activity. They identified 5 CPPs that improved eGFP signal by at least threefold, and 2 that did not, relative to unconjugated PMO. We used this published set of peptides to assess our model’s predictive accuracy.

### 2.3 Generation of sequence embeddings

Driven by the parallels between amino acid sequences and natural languages, scientists have utilized various techniques to represent peptides in a vectorized format. In this study, we explore two approaches – the W2V method and pre-trained LLMs – in the realm of CPP prediction.

#### 2.3.1 Word2Vec approach.

In recent years, the W2V technique [19] has been extensively utilized to generate word embeddings in the field of Natural Language Processing (NLP), demonstrating its robust performance. Among other studies, W2V has been employed to encode amino acid sequences, aiming to capture functional properties inferred from peptide sequences through contextual word learning [35].

W2V provides two alternative model architectures, namely Continuous Bag of Words (CBOW) and Skip-Gram (SG). After conducting several experiments, we found that the SG model generally outperformed the CBOW. In terms of its underlying algorithm, W2V processes and stores successive sequences of k-mer amino acids within a specified window of the sequence, treating each sequence as a distinct unit and representing it with a numerical vector. Our W2V model was designed to encode a sequence window of n amino acids into an (n−k+1)×S matrix, where *S* is the dimensionality of each vector representation.

We found that our model achieved better performance when we set the maximum amino acid sequence length (seqwin) to match the length of the longest peptide sequence among training datasets, i.e., the value 36, 61 and 27 for the CPP-Classification, Uptake-Efficiency and PMO-Delivery tasks, respectively. For the sequences with a length smaller than seqwin, we applied zero padding. Finally, regarding the implementation of W2V, we utilized the free open-source Python library Gensim [36]. We thoroughly examined the training parameters of W2V model, including the values of k-mer, vector size, number of training epochs, and window size (Table A in S1 Text).

Inspired by Kurata’s et al. [35] approach, we constructed three dataset-specific W2V dictionaries from the training and test datasets, one for each of the studied tasks, by adopting their sandwich structure concept. The training dataset is divided into subsets of positive and negative samples. Afterwards, the test dataset is shuffled and interleaved between them. This process resulted in our W2V dictionaries, from which our word embeddings were generated.

To examine whether the sandwich embedding strategy could introduce representational bias, we conducted an additional experiment in which the W2V model was trained exclusively on the training dataset (frozen setting) and subsequently kept fixed when encoding the *mlcpp* independent test dataset. The predictive performance under this setting remained essentially identical to the sandwich configuration (Accuracy: 0.8505 vs. 0.8473; MCC: 0.7301 vs. 0.7248; AUC: 0.9463 vs. 0.9460), indicating that the embedding strategy does not introduce bias from the evaluation data.

#### 2.3.2 Pre-trained LLMs approach.

Approaching the task from a different perspective, we carefully selected three pre-trained models based on the LLMs: Text-To-Text Transfer Transformer (T5) [37], Bidirectional Encoder Representations from Transformers (BERT) [17], and Evolutionary Scale Modeling (ESM-2) [18], to generate the embeddings of amino acid sequences. Similar to the usage of non-protein-based LLMs in language-based tasks, these models have been pre-trained on large protein databases comprising millions of sequences, where each amino acid is treated as a word and each sequence as a sentence, demonstrating their ability to reveal underlying semantic characteristics of peptides.

The selected models have been designed following the Masked Language Modeling pre-training approach, where a portion of the input tokens, 15% in our case, is randomly replaced with the special [MASK] token. Subsequently, the transformer architecture-based model is trained to predict the masked tokens, considering the surrounding unmasked tokens, and calculating the discrepancy between the predictions and the original targets. Once the loss has been computed, the model’s parameters are iteratively updated through backpropagation to minimize the loss function, resulting in meaningful embeddings [17]. Models’ hyperparameters are provided in Table B in S1 Text.

Regarding the T5 LLM, we utilized the ProtT5-XL-UniRef50 model [16], which has been pre-trained in a self-supervised manner on UniRef50, an extensive collection of 45 million protein sequences sourced from the UniProt database [38]. UniRef50 includes only sequences with less than 50% sequence similarity. T5 utilizes both encoder and decoder transformer models, whereas BERT exclusively adapts the encoder component.

In the case of the BERT LLM, we employed the ProtBERT model [16], which has been pre-trained similarly to ProtT5-XL-UniRef50 without any *a priori* human labelling. ProtBERT was mainly trained on UniRef100, a comprehensive dataset of 217 million protein sequences [38]. A significant distinction between ProtBERT and ProtT5-XL-UniRef50 models is that the former treats each involved sequence as a complete document without incorporating next sentence prediction [39].

Finally, regarding the ESM-2 LLM, we experimentally selected the esm2_t36_3B_UR50D model [40], which has been pre-trained on UniRef50 and fine-tuned on various databases including UniProt [38] and Protein Data Bank [41]. Additionally, the Unsupervised Data Augmentation technique is often used to enrich their data. Similar to T5 and BERT models, ESM-2 incorporates the transformer architecture and typically employs only the encoder component. Notably, ESM-2 was specifically designed for protein sequence analysis, capturing the structural characteristics of peptides [42].

### 2.4 Machine learning methods

To solve our classification problem for identifying CPPs, we employed three widely used conventional ML methods: SVM, RF and GB. Specifically, we used the Support Vector Classifier (SVC), a type of SVM designed for classification tasks, as implemented in the scikit-learn library [43]. An SVC aims to find the optimal hyperplane that separates data points of different classes by maximizing the margin between the hyperplane and the closest data points, known as support vectors. In contrast, both RF and GB are ensemble techniques that build multiple decision trees. RF constructs the decision trees in parallel and combines them for the final prediction, while GB builds them sequentially, where each tree corrects the errors of its predecessor.

Apart from the three traditional ML algorithms, we employed three deep learning (DL) architectures: Convolutional Neural Network (CNN), Transformer (TX), and Bidirectional Long Short-Term Memory (bLSTM). For all models, peptide sequences were first padded or truncated to a fixed length (i.e., seqwin), then represented into W2V embeddings to form a matrix of shape (seqwin×embedding dimension), which was used as input to the models. The CNN consisted of two convolutional layers to extract significant features, followed by two max-pooling layers to reduce spatial dimensions while preserving meaningful features. For TX, we utilized the encoder component to transform the amino acid sequences, while we set the number of attention heads and the depth of the neural network to 3 and 4, respectively. In the case of bLSTM, in order to identify hidden patterns of peptides, we selected an improved version of a Recurrent Neural Network (RNN) with memory cells and a gating structure to avoid vanishing gradient problems, while we set the number of expected features in the input data equal to the vector size of the W2V model and the number of features in the hidden state to 128.

#### 2.4.1 Construction of ML-based models.

Based on our encoding approach (W2V or Pre-trained LLM), we tested various models to identify the most promising for our studied tasks. We evaluated the performance of our models using both Cross-Validation (CV) and independent testing. In particular, we implemented a 10-fold CV for the CPP-Classification, a Jackknife Validation for the Uptake-Efficiency and a 3-fold CV for the PMO-Delivery [2,12].

During the development of CPP2Vec-GenSet, we explored several ways of constructing the negative class. For example, we tested datasets containing only moderate negatives, only hard negatives, or only natural negatives, in order to understand how the nature of the non-CPPs affected model behaviour. The most consistent results were obtained when all three types were combined, forming our proposed training dataset. For the CPP-Classification task, we then applied random undersampling of the majority (Non-CPP) class during training to maintain a balanced class distribution. In our experiments, the undersampled configuration produced more stable and generally better performance than training directly on the full 2,378 CPPs vs. 2,819 Non-CPPs dataset.

For the W2V approach, except for testing W2V parameters, i.e., seqwin, vector size, epochs, sg and window size, we evaluated our models for k-mer values within the range of 1–10 [35]. To explore the impact of dimensionality reduction on classification performance, Principal Component Analysis (PCA) was applied to the W2V embeddings, retaining 90%−99% of the variance. The effect of PCA was examined by comparing the performance of downstream classifiers trained on the reduced embeddings with those trained on the original, full-dimensional embeddings. Furthermore, for both of our encoding approaches, regarding RF, we executed the grid search that is proposed by Wolfe et al. [12] (Table 2), for the most significant parameters n_estimators, max_depth and max_features. In the case of SVC, we optimized the hyperparameters C, gamma, and the kernel type - to handle the trade-off between classification error and margin width, to control the influence of each training sample on the decision boundary, and to ensure that the model can appropriately capture potential nonlinear relationships in the data, respectively.

**Table 2:** Grid search to optimize RF classifier’s hyperparameters.

Proportion of Total Features	Number of Estimators	Maximum Tree Depth
0.75	50	20
0.5	50	20
0.25	50	20
0.5	10	20
0.5	250	20
0.5	1000	20
0.5	50	5
0.5	50	10

Hyperparameters tested for optimizing the RF classifier.

More details about hyperparameter optimization for both ML and DL methods are provided in Table C in S1 Text. The SVC, RF and GB classifiers were implemented using scikit-learn, while the CNN, TX and bLSTM models were executed through PyTorch [44].

### 2.5 Performance evaluation

To evaluate the prediction performance of the proposed models and compare them with state-of-the-art tools, we used six statistical measures, including Sensitivity (SE), Specificity (SP), Accuracy (ACC), Precision, Matthew’s Correlation Coefficient (MCC) and Area Under the Receiver Operating Characteristic Curve (AUC). The first five measures are calculated by the following equations:


$$\begin{array}{cc} Sensitivity & =\frac{TP}{TP+FN}\\ Specificity & =\frac{TN}{TN+FP}\\ Accuracy & =\frac{TP+TN}{TP+TN+FN+FP}\\ Precision & =\frac{TP}{TP+FP}\\ MCC & =\frac{TP\times TN-FP\times FN}{\sqrt{\left(TP+FN)(TP+FP)(TN+FP)(TN+FN\right)}} \end{array}$$

where TP, TN, FP, and FN denote the numbers of true positive, true negative, false positive and false negative, respectively. Finally, the AUC metric is computed by integrating the ROC curve, the graphical representation of SE against 1 – SP.

## 3 Results

In this section, we outline the approach that we followed to select our proposed models and we present their overall performance across all tasks and datasets (Table 3). Furthermore, we include a detailed task-centered comparison of CPP2Vec against state-of-the art tools, including a visualized performance of both CPP2Vec and CPP2LLM on each task separately. Finally, we provide six radar charts to depict the overall predictive performance of each model across all datasets, highlighting their strengths and weaknesses.

**Table 3:** Performance metrics of CPP2Vec and CPP2LLM across tasks and datasets.

Task	Dataset	SE	SP	ACC	MCC	AUC	Precision
**CPP2Vec**							
**CPP Classification**	Validation	0.910	0.950	0.931	0.862	0.973	0.939
	Test: *kelm*	0.799	0.941	0.870	0.748	0.934	0.932
	Test: *mlcpp*	0.988	0.755	0.871	0.764	0.953	0.801
**Uptake Efficiency**	Validation	0.754	0.679	0.716	0.434	0.727	0.701
	Test	0.444	0.756	0.625	0.211	0.707	0.571
**PMO Delivery**	Validation	0.719	0.858	0.782	0.589	0.839	0.800
	Test	0.800	1.000	0.857	0.730	1.000	1.000
**CPP2LLM**							
**CPP Classification**	Validation	0.886	0.928	0.909	0.816	0.962	0.913
	Test: *kelm*	0.795	0.884	0.840	0.682	0.855	0.873
	Test: *mlcpp*	0.983	0.695	0.839	0.709	0.956	0.764
**Uptake Efficiency**	Validation	0.610	0.695	0.652	0.306	0.698	0.667
	Test	0.552	0.731	0.656	0.288	0.662	0.600
**PMO Delivery**	Validation	0.700	0.889	0.781	0.624	0.890	0.861
	Test	0.534	1.000	0.667	0.512	0.983	1.000

SE: Sensitivity, SP: Specificity, ACC: Accuracy, MCC: Matthews Correlation Coefficient, AUC: Area Under Curve.

### 3.1 Selection of CPP2Vec and CPP2LLM

#### 3.1.1 CPP2Vec.

To determine which W2V and ML model provides the best results for each task, we executed a series of experiments, aiming to find the optimal value for each one of the involved parameters. Selected results are provided in S2 Text. In the first step, we focused on the W2V model optimization. We started by stabilizing the ML model and experimenting with vector size, epochs, sg and window parameters. For instance, in Table 4 we provide a selection of representative results from our experiments on W2V model hyperparameter optimization for the PMO-Delivery task. We worked in a similar way for each one of the three studied tasks.

**Table 4:** Selected representative results from our W2V model hyperparameter optimization experiments for the PMO-Delivery task.

W2V Parameters	Validation		Test	
	ACC	MCC	ACC	MCC
27, 300, 8, 1, 20	0.748	0.502	0.619	0.449
27, 100, 12, 1, 20	0.780	0.549	0.619	0.449
27, 100, 20, 0, 20	0.670	0.323	0.571	0.387
27, 100, 20, 1, 10	0.766	0.523	0.571	0.401
**27, 100, 20, 1, 20**	**0.782**	**0.560**	**0.857**	**0.676**

W2V Parameters are depicted as: (seqwin, size, epochs, sg, window), where sg = 1 indicates Skip-Gram approach, while sg = 0 indicates Continuous Bag of Words. ACC: Accuracy, MCC: Matthews Correlation Coefficient.

Subsequently, we attempted to identify the most promising ML algorithms. For the CPP-Classification task we tested various ML algorithms, including RF, SVC, GB, TX, bLSTM and CNN. In Table 5 we present some selected results, that led us to select SVC with C = 5.0 as the optimal model.

**Table 5:** Performance metrics of ML Models across validation and test datasets.

ML Model	Validation		Test: *kelm*		Test: *mlcpp*	
Parameters	ACC	MCC	ACC	MCC	ACC	MCC
RF	0.904	0.807	0.839	0.691	0.873	0.764
GB	0.913	0.826	0.813	0.634	0.854	0.716
TX	0.836	0.692	0.724	0.455	0.799	0.623
bLSTM	0.850	0.711	0.833	0.689	0.822	0.663
CNN	0.852	0.717	0.719	0.442	0.789	0.617
**SVC**	**0.931**	**0.862**	**0.870**	**0.746**	**0.876**	**0.771**

RF(max_features = 0.5, max_depth = 20, n_estimators = 50), SVC(C = 5.0, kernel = ‘poly’, probability = True), GB(n_estimators = 100, learning_rate = 0.1, max_depth = 4), TX (n_layers = 4, n_heads = 8, d_model = 128, d_ff = 256, input_dim = 7000, epochs = 8, lr = 0.001, batch_size = 64), bLSTM (hidden_dim = 128, num_layers = 3, bidirectional = True, input_dim = 7000, epochs = 8, learning_rate = 0.001, batch_size = 64), CNN(kernel_size = 3, input_dim = 7000, epochs = 8, learning_rate = 0.001, batch_size = 64).

For the Uptake-Efficiency task, we executed some initial trials with SVC models, based on the CPP-Classification results. We conducted the grid search proposed by Wolfe et al. [12] (Table 2) and we compared the results across models. We observed that the SVC with C = 10.0 outperformed the best RF model, achieving a validation accuracy of 71.7% compared to 67.9%, respectively, leading us to its selection as the optimal classifier.

Finally, as the accuracy of the Uptake-Efficiency task model remained low even after optimizing the model’s parameters, we decided to use the 64-peptides dataset as a training dataset to predict PMO-Delivery efficiency. We initially found the best values for the W2V model and then we executed the same grid search. The proposed W2V model values for CPP-Classification, Uptake-Efficiency and PMO-Delivery tasks are depicted in Table 6.

**Table 6:** Overview of the proposed W2V model parameters per task.

W2V Parameter	CPP Classification	Uptake Efficiency	PMO Delivery
**kmer**	2	3	1
**seqwin**	36	61	27
**vector size**	200	300	100
**epochs**	8	12	20
**sg**	1	1	1
**window**	20	20	20

Values correspond to the optimal W2V hyperparameters selected per task.

The only difference is that in this case we repeated our experiments for the best two models, as datasets consist of a small number of sequences. The average metrics for both StratifiedKFold and KFold cross-validators [43] can be found in the Tables D and E in S1 Text. The proposed ML models for all tasks are depicted at Table 7.

**Table 7:** Proposed ML models per task of CPP2Vec.

Task	ML Model
CPP-Classification	SVC (C = 5.0, kernel = ’poly’, prob = True)
Uptake-Efficiency	SVC (C = 10.0, kernel = ’rbf’, prob = True)
PMO-Delivery	RF (max_features = 0.5, max_depth = 20, n_estimators = 250)

#### 3.1.2 CPP2LLM.

For each of the tasks we studied, i.e., CPP-Classification, Uptake-Efficiency, and PMO-Delivery, we conducted 12 experiments per LLM (ProtT5, ProtBERT and ESM-2). Specifically, for each task and each LLM we ran 3 experiments with SVC (C = 1.0, C = 5.0 and C = 10.0), 1 experiment with GB (n_estimators = 100, learning_rate = 0.1, max_depth = 4) and 8 experiments with the RF classifiers of the proposed grid search. By taking into consideration the results and their running times we selected the LLM and ML models that are presented in Table 8.

**Table 8:** Proposed LLM and ML models per task of CPP2LLM.

Task	LLM Model	ML Model
CPP-Classification	BERT	RF (max_features = 0.5, max_depth = 20, n_estimators = 1000)
Uptake-Efficiency	ESM-2	SVC (C = 10.0, kernel = ‘rbf’, prob = True)
PMO-Delivery	ESM-2	RF (max_features = 0.25, max_depth = 20, n_estimators = 50)

### 3.2 Visualization of CPP2Vec embeddings

To validate the effectiveness of our proposed models in distinguishing between the studied classes, i.e., CPP/non-CPP, High/Low uptake efficiency, and ≥ 3-fold/ < 3-fold improvement, we visualized the learned W2V embeddings in 2D space using supervised Uniform Manifold Approximation and Projection (UMAP) [45] and PCA [46].

**Fig 3:**
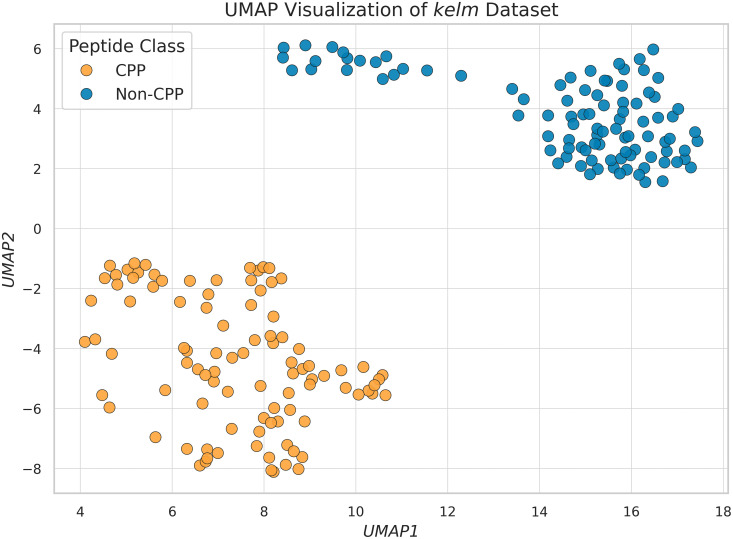
Supervised UMAP visualization of the *kelm* dataset. CPPs and Non-CPPs are depicted in orange and blue, respectively. The clear distinction between the two classes highlights the representation power of CPP2Vec.

In Fig 3, the UMAP plot for the *kelm* dataset reveals clearly separated clusters corresponding to each class, indicating that the embeddings capture meaningful structural and functional patterns in the sequences. Similar independent visualizations for the *mlcpp*, 64-peptides, and 7-novel-sequences datasets are provided in S1 Text, specifically in Figs A-C, respectively, showing well-defined class boundaries.

To rigorously evaluate the generalization capability of the learned embeddings, we additionally performed UMAP embedding using a single model fitted on the training dataset for each task, and projected the validation folds and independent test sets onto this shared embedding space. In this configuration, the embeddings retain inter-class separation while allowing direct comparison across splits. UMAP plots for the training and test datasets, along with one representative validation fold for the CPP Classification task, are provided in S1 Text (Figs D-G). Similar plots for the Uptake Efficiency and PMO Delivery tasks can be found in S1 Text (Figs H-J and Figs K-M, respectively).

Cluster statistics, including silhouette scores, mean intra- and inter-class distances, inter/intra ratios, and Mann–Whitney U test *p*-values, were computed for the training, validation, and test datasets. Statistics for the CPP Classification task are presented in Table 9, while the corresponding statistics for the Uptake Efficiency and PMO Delivery tasks can be found in S1 Text, Tables F and G, respectively.

**Table 9:** Cluster statistics of W2V embeddings for the CPP Classification task across training, validation, and independent test sets.

Dataset	Silhouette Score	Mean Intra-class Distance	Mean Inter-class Distance	Inter/Intra Ratio
**CPP2Vec-GenSet**	0.6053	6.2131	15.9797	2.572
**Validation (mean)**	0.5625	6.6278	15.6198	2.361
***kelm***	0.3220	8.3090	12.6922	1.528
***mlcpp***	0.3762	7.3300	12.8136	1.748

Cluster statistics were computed from the W2V embeddings projected via UMAP fitted on the CPP2Vec-GenSet training dataset for the CPP Classification task. Silhouette score measures the separation of clusters; mean intra-class and inter-class distances quantify within-class compactness and between-class separation; inter/intra ratio indicates the relative class separation. Validation statistics are averaged across the 10 cross-validation folds.

For the CPP Classification task, the W2V embeddings demonstrate strong and well-separated clusters in the training set (CPP2Vec-GenSet silhouette = 0.6053; inter/intra ratio = 2.57), which is largely preserved across cross-validation folds (mean silhouette = 0.5625; ratio = 2.36). Independent datasets (*kelm* and *mlcpp*) maintain consistent inter-class separation (silhouette = 0.3220 – 0.3762; inter/intra ratio = 1.53 – 1.75), indicating that CPP2Vec captures sequence features that remain discriminative across external datasets when biologically coherent class boundaries exist, supporting reliable differentiation between CPP and non-CPP sequences.

For the Uptake Efficiency task, the degree of geometric separation observed is substantially lower than that of the CPP Classification task and the PMO Delivery training dataset (64-peptides silhouette = 0.5932). This reduced separation is already noticeable in the training dataset itself (CPPsite3_Gautam silhouette = 0.2522), indicating that the inherent class structure in this dataset is less well-defined in sequence space. This suggests that uptake efficiency may not form sharply separable clusters based solely on primary sequence features, reflecting poorly defined class boundaries, in contrast to CPP Classification.

This observation likely reflects broader challenges in the field rather than limitations of the embedding approach. Quantitative uptake measurements are influenced by experimental variability, heterogeneous biological systems, and partially understood internalization mechanisms. As a result, peptides categorized as “high” and “low” uptake may exhibit overlapping sequence characteristics, reducing geometric separability in embedding space. Such effects are compounded by differences in assay conditions, cell types, and peptide concentrations, which are not encoded by current sequence-based state-of-the-art computational models.

These findings further emphasize that advancing functional prediction performance will likely require computational frameworks that incorporate relevant biological context, including tissue specificity and dose-dependent effects, beyond sequence-derived features alone.

**Fig 4:**
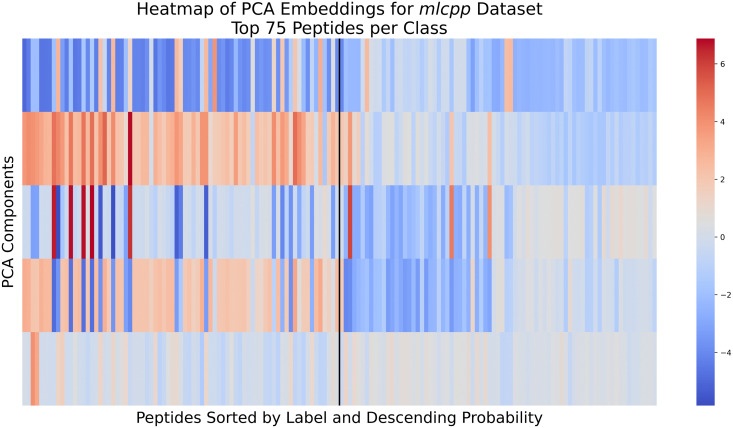
Heatmap of the first five PCA Components for the *mlcpp* dataset. Top 75 peptides per class are shown, grouped by labels from CPPs to Non-CPPs and sorted by descending predicted probability scores. The black vertical line illustrates the boundary between classes.

To gain insight into how sequence-derived features inform functional classification, we explored the main patterns emerging in the feature space. In Fig 4 we present the heatmap of the first five PCA components across peptides for the *mlcpp* dataset, showing the Top 75 peptides per class, grouped by class labels and sorted by descending predicted probability score. Differences in color intensity between classes suggest that these PCA components can effectively distinguish the peptides. Within each class, the existence of color patterns among peptides suggest shared characteristics, while some PCA components show greater variability, indicating that there are features that contribute less heavily to class distinction. The observed intra-class diversity, including peptides with unique component compositions, highlights the need for a training dataset like CPP2Vec-GenSet, which was designed with the aim of capturing the full spectrum of peptide heterogeneity. The heatmaps for the other test datasets are provided in S1 Text (Figs N-P).

### 3.3 Comparison of CPP2Vec with state-of-the-art tools

#### 3.3.1 CPP-classification.

**Fig 5:**
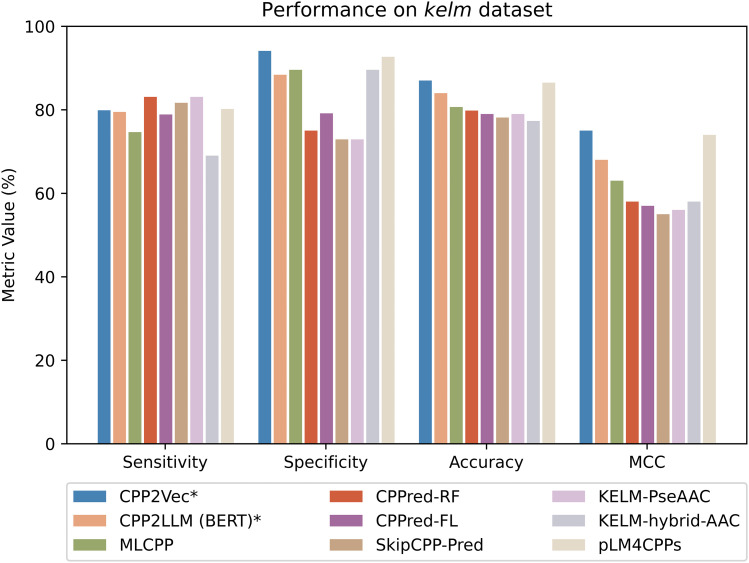
Comparison of CPP2Vec and CPP2LLM with state-of-the-art tools on *kelm* dataset. Our proposed models are marked with an asterisk (*). Each bar represents the mean performance across the 10-fold CV. The selected protein-based LLM of CPP2LLM is shown in parentheses.

**Fig 6:**
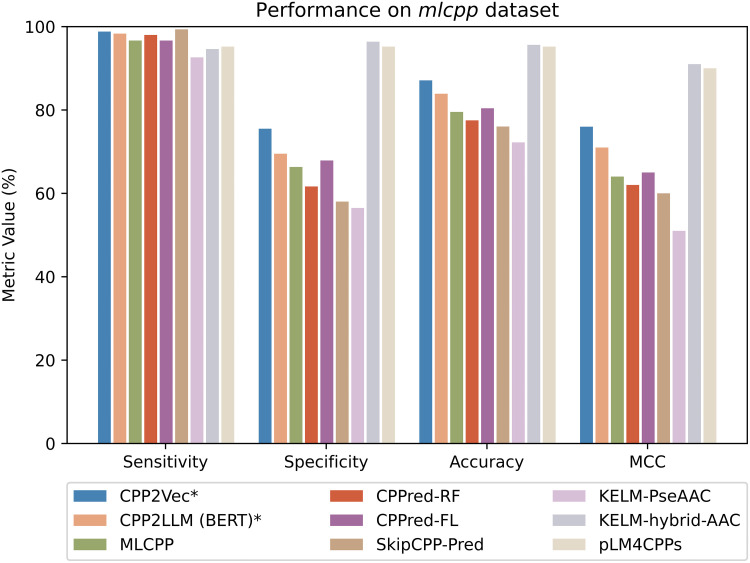
Comparison of CPP2Vec and CPP2LLM with state-of-the-art tools on *mlcpp* dataset. Our proposed models are marked with an asterisk (*). Each bar represents the mean performance across the 10-fold CV. The selected protein-based LLM of CPP2LLM is shown in parentheses.

To comprehensively evaluate the performance of CPP2Vec, we compared it with state-of-the-art CPP prediction tools across two independent benchmark datasets, *kelm* and *mlcpp*, which were also utilized in the comparative analysis conducted by Su et al. [2] A summary of the results for selected models is presented in Figs 5 and 6, corresponding to the *kelm* and *mlcpp* datasets, respectively, while a complete comparison with all available models is provided in S1 Text (Tables H and I).

On the *kelm* dataset, CPP2Vec achieved an accuracy of 87% and a MCC score of 0.75 outperforming all competing tools, including advanced ensemble and transformer-based methods. CPP2Vec achieved high performance on both classes, with 79.9% sensitivity and 94.1% specificity, demonstrating its reliability in identifying CPP and non-CPP sequences. These results highlight the discriminative capability of CPP2Vec in learning biologically meaningful peptide representations that translate effectively into predictive performance.

On the *mlcpp* dataset, CPP2Vec sustained robust generalization, achieving a SE of 98.8%, SP of 75.5%, accuracy of 87.1%, and MCC of 0.76. Despite differences in dataset composition and feature representation, CPP2Vec consistently ranked among the top performers, confirming its adaptability and resilience across heterogeneous data sources. The high SE observed on this dataset underscores its capability to capture diverse CPP sequence patterns without sacrificing predictive reliability.

Beyond external evaluation, CPP2Vec also exhibited exceptional internal validation performance. Under a 10-fold CV, the model achieved SE, SP, and accuracy values of 91%, 95%, and 93.1%, respectively, with an MCC of 0.862, AUC of 97.3%, and precision of 93.9%. This combination of high accuracy, balanced SE–SP tradeoff, and almost perfect AUC demonstrates CPP2Vec’s predictive stability and resistance to overfitting.

CPP2Vec achieved these results with remarkable computational efficiency: training, prediction and evaluation (10-fold CV and independent dataset testing) required only 80 minutes on the *kelm* dataset, using a CPU-only system with a single-socket Intel Xeon Gold 6148 CPU @ 2.40 GHz (20 cores / 40 threads) and 64 GB RAM, compared to approximately 16 hours for CPP2LLM, which was run on the same hardware and evaluation protocol and serves as a representative example of protein LLM-based approaches in the field. This corresponds to a twelvefold reduction in runtime while maintaining comparable or superior predictive performance.

These findings position CPP2Vec as a robust and scalable CPP prediction framework that combines high predictive accuracy, strong generalization across datasets, and exceptional efficiency, achieving state-of-the-art performance with markedly reduced computational cost.

#### 3.3.2 Uptake-efficiency.

**Fig 7:**
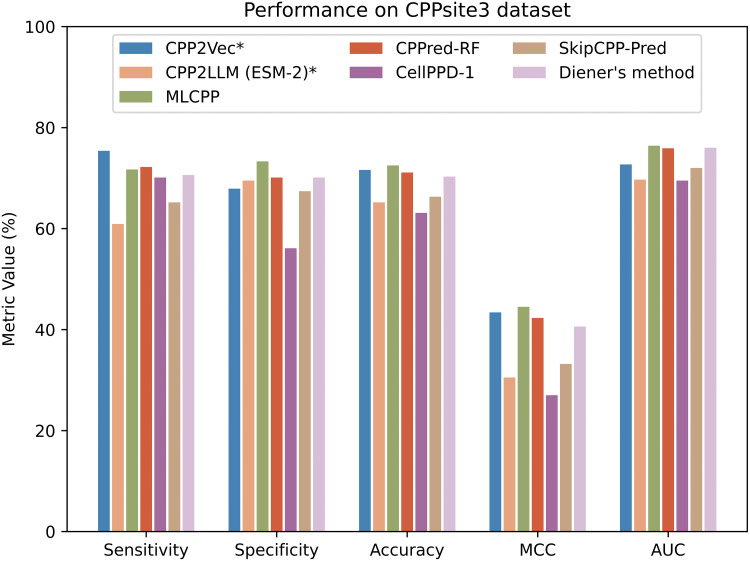
Comparison of CPP2Vec and CPP2LLM with state-of-the-art tools on CPPsite3_Gautam dataset. Our proposed models are marked with an asterisk (*). Each bar represents the mean performance across the Jackknife Validation. The selected protein-based LLM of CPP2LLM is shown in parentheses.

To evaluate the performance of CPP2Vec at the Uptake-Efficiency task, we compared the results of jackknife validation with the metrics of state-of-the-art predictors derived from Manavalan’s et al. [10] study, on the CPPsite3_Gautam dataset [7] (Fig 7).

CPP2Vec scores the highest SE of 75.4% among all the compared methods, highlighting its ability to accurately detect peptides with high uptake efficiency. In terms of overall ACC, CPP2Vec scores 71.6%, closely trailing MLCPP (72.5%), while outperforming other models such as SkipCPP-Pred (66.3%), CellPPD (63.1%), and CPPred-RF (71.1%). For MCC, CPP2Vec achieves the second-best performance with a score of 0.434, following MLCPP (0.445), and ahead of CPPred-RF (0.423), Diener’s method (0.406), SkipCPP-Pred (0.332), and CellPPD (0.270). Finally, CPP2Vec reaches an AUC of 72.7% and a SP of 67.9%, keeping it in a competitive range, although MLCPP exceeds it with an AUC of 76.4% and a SP of 73.3%.

#### 3.3.3 PMO-delivery.

**Fig 8:**
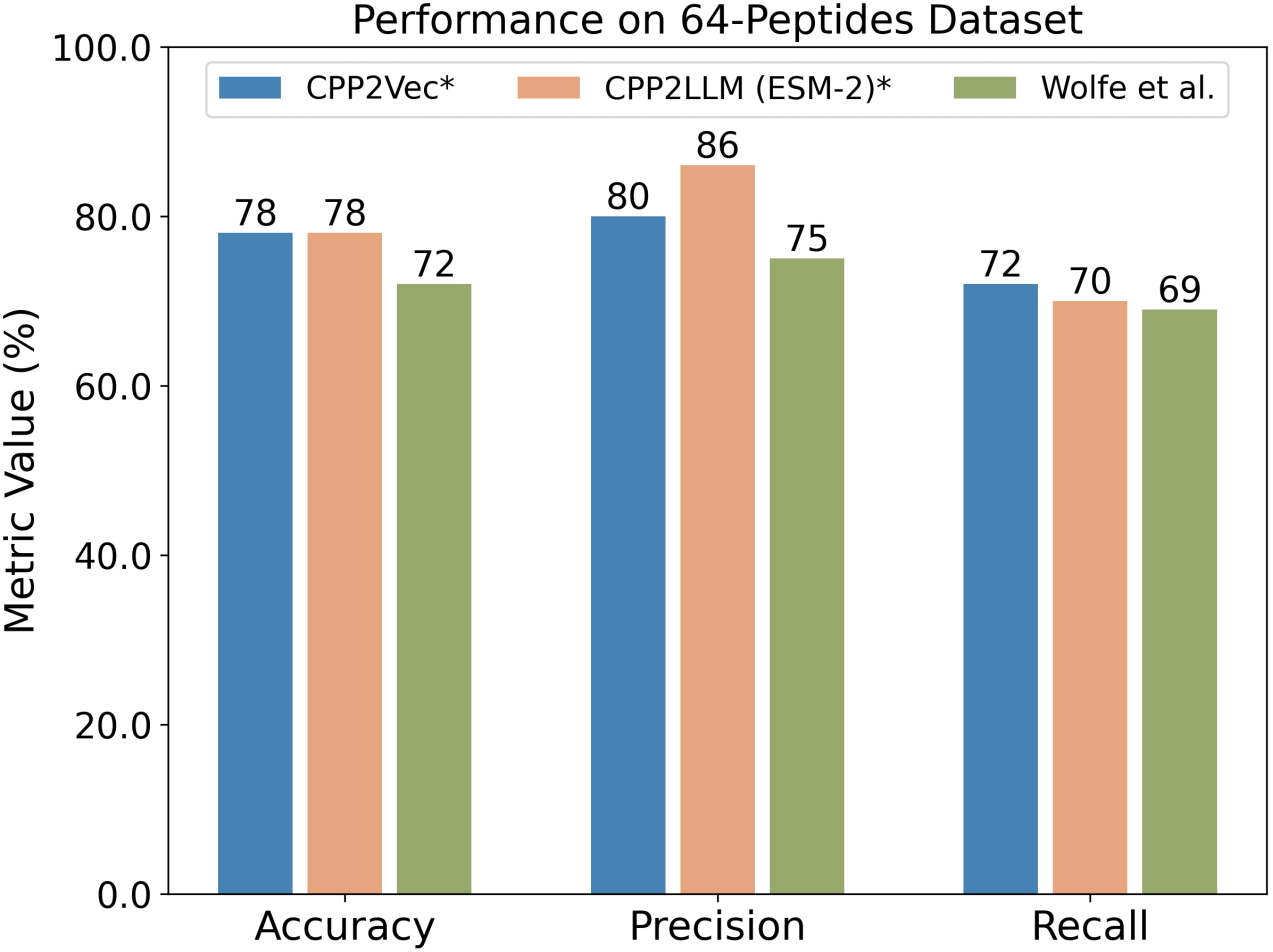
Comparison of CPP2Vec and CPP2LLM with Wolfe’s et al. [12] method on 64-peptides dataset. Our proposed models are marked with an asterisk (*). Each bar represents the mean performance across the 3-fold CV. The selected protein-based LLM of CPP2LLM is shown in parentheses.

As none of the current state-of-the-art CPP prediction tools provide a model for detecting peptides that could enhance PMO-Delivery across the plasma membrane, we compared the performance of CPP2Vec to the method that Wolfe et al. [12] proposed in their study, on the 64-peptides dataset (Fig 8). Specifically, CPP2Vec showed an accuracy of 78%, a precision of 80% and a recall of 72%, which are 6%, 5% and 3% higher than Wolfe’s et al. method, respectively.

#### 3.3.4 Overall predictive performance.

**Fig 9:**
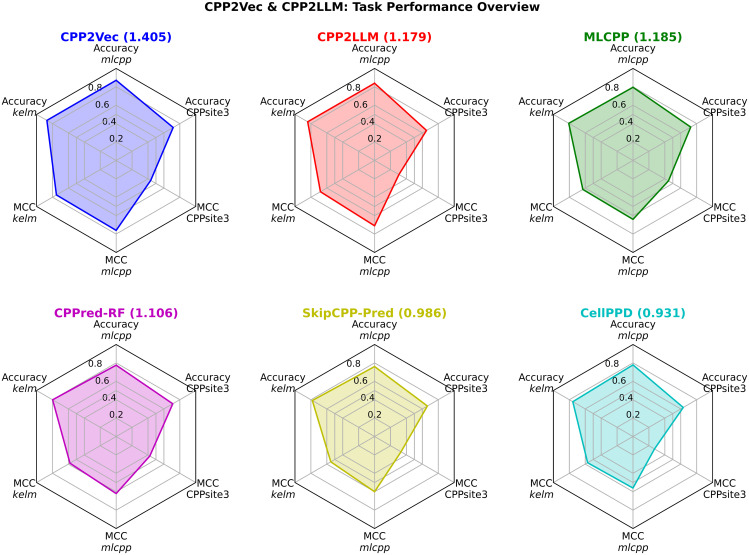
Depiction of the overall predictive performance per model. Each polygon represents a tool, i.e., MLCPP, CPPred-RF, SkipCPP-Pred, CellPPD, as well as our proposed models, CPP2Vec and CPP2LLM. The top three edges represent the accuracy of the models on the *kelm*, *mlcpp*, and CPPsite3_Gautam datasets, while the bottom edges denote their respective MCC scores. In general, larger areas indicate better performance (values in parentheses).

Fig 9 illustrates the predictive performance of each model across the CPP-Classification and Uptake-Efficiency tasks. We present six radar charts where each polygon corresponds to a specific tool, including MLCPP, CPPred-RF, SkipCPP-Pred, CellPPD, as well as our proposed models, CPP2Vec and CPP2LLM. The top three edges of each polygon represent the accuracy of the models on the *kelm*, *mlcpp*, and CPPsite3_Gautam datasets, while the bottom three edges denote their corresponding MCC scores.

CPP2Vec demonstrates the highest overall predictive performance, reflecting its robust and stable outcomes across all datasets. Notably, it achieves a balanced and consistently high performance across diverse predictive scenarios, underscoring its strong generalization capability. This reliability highlights CPP2Vec’s potential as a dependable tool for CPP prediction in a variety of contexts.

## 4 Case study

We selected manually from the available literature 14 peptides that have been tested for their PMO-Delivery efficiency on mdx mice [22]. The mdx mouse is a famous model that was discovered in 1984 and it has been extensively used to study DMD [20,21]. The mdx mouse carries a point mutation in exon 23 of the DMD gene, where a Cytosine (C) is replaced by a Thymine (T), converting a Glutamine codon (CAA) into a premature stop codon (TAA) [47]. This nonsense mutation results in a truncated, non-functional dystrophin protein. The symptoms are not as severe as in humans; however, they share a lot of similarities in their DMD genes making them a proper model for *in vitro* and *in vivo* trials.

Details about the 14 selected peptides are provided in Table 10. The first four peptides, namely muscle-specific peptide (MSP), trans-activating transcriptional activator (TAT), adeno-associated virus 6 (AAV6) and AAV8, have been used by Yin et al. [48] in 2007, administering a single intramuscular injection (5μg IM) in the tibialis anterior (TA) muscles of 2-month-old mdx mice. Interestingly in their study they used PNAs instead of PMOs, for stronger stability. In 2008, Matthew Wood et al. [49] tested naked B-peptide and its conjugation to PMO. A single 5μg IM injection into the TA muscle showed promising local exon skipping, while in the same study, systematic delivery of B–PMO induced up to 55% dystrophin restoration in skeletal muscles, including the TA. Finally, in 2008 Jearawiriyapaisarn et al. [51] constructed a series of peptides with improved, not only muscle, but also cardiac exon skipping activity for DMD treatment [52]. This was the first study where exon skipping was detected in cardiac muscle, reaching 15% of normal levels of dystrophin protein.

**Table 10:** Amino acid sequences and properties of the 14 peptides of the DMD dataset.

Name	Amino Acid Sequence	Length	Number of Arginines	Western Blot
MSP	ASSLNIA	7	0	3%^1^
TAT	YGRKKRRQRRRP	12	6	3%^1^
AAV6	TVAVNLQSSSTDPATGDVHVM	21	0	3%^1^
AAV8	IVADNLQQQNTAPQIGTVNSQ	21	0	3%^1^
B	RXRRBRRXRRBRXB	14	8	55%^2^
Pip5e	RXRRBRRXRILFQYRXRBRXRB	22	10	88%^3^
Pip5f	RXRRBRRXRILFQYRXRXRXRB	22	10	63%^3^
Pip5h	RXRRXRILFQYRXRRXR	17	8	38%^3^
Pip5j	RBRRXRRBRILFQYRBRXRBRB	22	10	90%^3^
Pip5k	RBRRXRRBRILFQYRXRBRXRB	22	10	63%^3^
Pip5l	RBRRXRRBRILFQYRXRRXRB	21	10	70%^3^
Pip5m	RBRRXRRBRILFQYRXRBRXB	21	9	38%^3^
Pip5n	RXRRBRRXRILFQYRXRRXRB	21	10	78%^3^
Pip5o	RXRRBRRXRILFQYRXRBRXB	21	9	40%^3^

Along with the amino acid sequence, the table includes basic sequence properties – length and number of arginine residues – as well as Western Blot measurements reflecting each peptide’s efficiency to induce exon skipping. Dystrophin restoration levels are expressed as a percentage relative to control sample. Western Blot data sourced from: 1. Yin et al. [48], 2008; 2. Yin et al. [49], 2008; 3. Yin et al. [50], 2011.

Building on this, Yin et al. [50] later developed and systematically evaluated a panel of Pip5 peptides conjugated to PMOs, optimizing features such as charge, sequence length, and arginine-rich content to enhance delivery efficiency. Several of these variants showed markedly improved exon-skipping activity across skeletal muscles, including the TA, as well as cardiac muscle, demonstrating significant advances in peptide-mediated delivery for effective DMD therapy. The Western blot data summarized in the table originate from this study, where the authors systematically assessed the exon-skipping efficacy of the various Pip5 peptide–PMO conjugates in muscle tissues.

As part of the CPP2Vec model construction, β-alanine (B) was substituted with α-alanine (A) and 6-aminohexanoic acid (X) with lysine (K), ensuring that all sequences consisted of standard amino acids and were compatible with our model framework.

**Fig 10:**
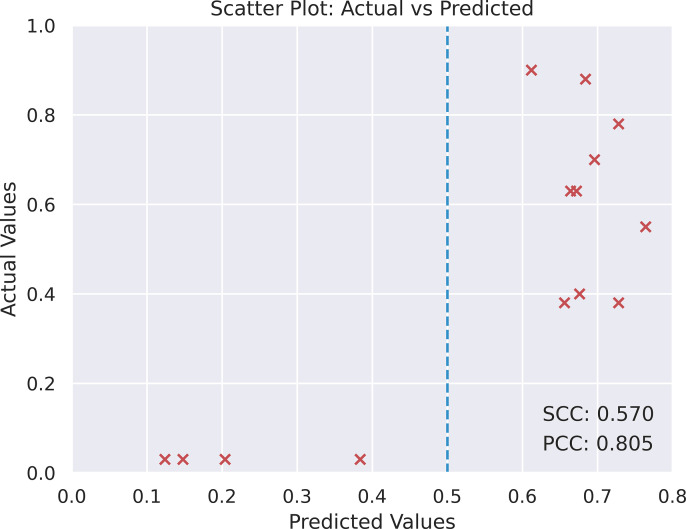
Scatter plot of the predicted vs actual values on the DMD dataset. Each red cross represents one of the 14 peptides. The vertical dashed line on 0.5 illustrates the model’s ability to accurately distinguish efficient peptides from inefficient ones.

Finally, we tested our PMO-Delivery model on the DMD dataset, to thoroughly evaluate its performance, assessing its practical applicability and effectiveness in handling real case challenges. We calculated the Spearman’s correlation coefficient (SCC) and Pearson’s correlation coefficient (PCC) between the predicted probabilities and the Western Blot percentages, and we depicted the model’s performance using a Scatter Plot (Fig 10). We clearly delineated between efficient and inefficient peptides, highlighting the ability of CPP2Vec to accurately predict peptide delivery efficacy.

## 5 Discussion

In this study, we introduced CPP2Vec, an ML-based CPP prediction tool that utilizes the W2V technique to generate the amino acid sequence embeddings, aiming to capture hidden peptide patterns that underlie CPP functionality. To further enhance the learning capacity of our framework, we constructed CPP2Vec-GenSet, a novel hybrid dataset integrating both experimentally validated CPPs and synthetically generated peptide variants. This enriched dataset provides a more diverse and biologically relevant training foundation, which is particularly valuable given the high cost and labor intensity of wet-lab experiments. By combining robust embeddings with an expanded dataset, CPP2Vec enables the efficient *in silico* identification of novel CPPs and preliminary estimation of their uptake potential. Building on this foundation, we developed three task-specific ML classifiers and additionally explored three DL architectures to address CPP Classification, Uptake Efficiency, and PMO Delivery.

Our proposed models were extensively evaluated and compared against state-of-the-art tools, achieving significant predictive performance. A key advantage of CPP2Vec is its ability to deliver accurate and robust predictions without requiring any prior feature engineering and with exceptionally fast runtime, relying only on the amino acid sequence of peptides. As expected, RF and SVC models outperformed DL models, likely reflecting the limited size of available training datasets. Consistent with prior work, CPP2Vec achieved moderate performance on the uptake-efficiency task, highlighting the challenges posed by the complex and partially understood internalization mechanisms of CPPs and the limited availability of high-quality quantitative data.

It is also important to consider the characteristics of widely used benchmark datasets when interpreting comparative performance. In the *mlcpp* dataset, negative samples were constructed by excluding peptides exhibiting sequence similarity to known CPPs, a strategy likely intended to minimize potential false negatives. While this design choice improves label reliability, it may also reduce ambiguity near the decision boundary and thereby simplify the classification task. Nevertheless, *mlcpp* has been consistently employed as a standard benchmark across prior state-of-the-art predictors, enabling fair and transparent comparisons.

To eliminate potential dataset-specific bias and ensure robust assessment of generalization, we additionally evaluated CPP2Vec on the *kelm* independent dataset. Notably, whereas several existing methods demonstrate performance variability across independent benchmarks, CPP2Vec maintains consistently strong predictive performance on both datasets. This cross-dataset stability indicates that the observed improvements are not attributable to the characteristics of any single benchmark, but rather reflect genuine generalization capability arising from the diverse and biologically relevant training strategy employed in this study. These observations provide further confidence in the robustness of our framework as a generalizable predictive tool.

Despite these advances, a notable limitation shared by all existing tools, including CPP2Vec, is the absence of tissue-specific predictive capability. Current computational models generally treat CPP activity as a context-independent property, whereas experimental evidence indicates that cellular and tissue environments can substantially influence uptake and functional delivery. For example, the recent DG9 study [53] demonstrated markedly different PMO delivery outcomes in muscle versus cardiac tissue, underscoring the importance of tissue-dependent interactions. Similarly, dose-dependent effects are not captured by existing models, despite the well-documented influence of concentration on uptake efficiency. Incorporating both tissue-specific context and dose-response behavior into future computational frameworks will be essential to achieve more accurate and physiologically meaningful CPP predictions.

Approaching the CPP prediction task from a different angle, we explored using protein-based LLMs to generate contextualized embeddings. Although this strategy achieved performance comparable to CPP2Vec, it required significantly higher computational resources, emphasizing the practical advantage of CPP2Vec, that not only delivers competitive accuracy, but also remains fully reproducible and adaptable to new datasets and tasks. Unlike LLM-based models, which are computationally prohibitive to retrain and often function as closed systems, CPP2Vec can be efficiently implemented on standard hardware. It can be retrained using the entire dataset, a subset of it, or by adding new peptides, enabling its integration into bioinformatics and therapeutic discovery workflows.

Finally, we developed a specialized PMO-Delivery model to predict whether a peptide could enhance the delivery of a PMO-complex into the cell relative to its naked form. In a case study focused on DMD, our model demonstrated strong predictive performance and highlighted CPP2Vec’s potential to guide the discovery of novel therapeutically relevant CPPs. Beyond prediction, insights from the model could inform experimental prioritization, accelerate peptide design, and support the development of more effective peptide-conjugated therapeutics.

In summary, CPP2Vec represents a robust, generalizable, and computationally efficient framework for CPP prediction, combining high predictive performance with minimal computational overhead and no requirement for manual feature engineering. By integrating a novel hybrid dataset, supporting multiple predictive tasks, and demonstrating translational relevance through the DMD case study, CPP2Vec provides a practical and scientifically rigorous tool for guiding peptide discovery and development.

## 6 Conclusion

In this research, we introduced CPP2Vec, an ML-based tool for CPP prediction that utilizes the W2V approach to encode amino acid sequences, aiming to uncover underlying biologically meaningful peptide patterns. We developed three specialized models, each tailored to excel in one of the following tasks: CPP Classification, Uptake Efficiency and PMO Delivery. A key advantage of CPP2Vec is its ability to eliminate manual, task-specific feature engineering by automatically learning meaningful representations – while maintaining exceptionally fast runtime.

To strengthen model training, we constructed CPP2Vec-GenSet, a hybrid dataset integrating experimentally validated and synthetically generated peptides, providing a rich and biologically relevant foundation. Comprehensive benchmarking against state-of-the-art tools, together with a targeted DMD case study, highlights CPP2Vec’s strong and consistent predictive performance. Its data-driven architecture, fast runtime, and ability to generalize across biologically distinct prediction tasks position CPP2Vec as a powerful and reproducible resource for accelerating CPP discovery. Given the substantial cost and labor associated with experimental screening, CPP2Vec offers a practical and effective *in silico* alternative capable of supporting experts in diverse therapeutic contexts and facilitating the development of next-generation delivery peptides.

## Supporting information

S1 Text Supplementary material. **Table A.** Word2Vec hyperparameter tuning for CPP2Vec. **Table B.** Hyperparameters of the pre-trained CPP2LLM models. **Table C.** Hyperparameter search regions per machine learning or deep learning method. **Table D.** The average metrics for StratifiedKFold. **Table E.** The average metrics for KFold. **Fig A.** UMAP visualization of *mlcpp* dataset. With orange are represented the CPPs while with blue the Non-CPPs. **Fig B.** UMAP visualization of 64-peptides dataset. With orange are noted the peptides with high uptake efficiency, while with blue those with low uptake efficiency. **Fig C.** UMAP visualization of 7-novel-sequences dataset. With orange are depicted the peptides with a ≥ 3-fold improvement in eGFP fluorescence compared to unconjugated PMOs, while with blue those with <3-fold improvement. **Fig D.** UMAP visualization of CPP2Vec-GenSet for the CPP Classification task. The UMAP model was fitted on this training dataset (i.e., CPP2Vec-GenSet) and the embeddings of the validation and independent test sequences were projected onto the same embedding space. CPPs are depicted in orange, while Non-CPPs are shown in blue. **Fig E.** UMAP visualization of the *kelm* independent test dataset for the CPP Classification task. The UMAP model was fitted on the CPP2Vec-GenSet training dataset, and the test embeddings were projected onto the same embedding space. CPPs are depicted in orange, while Non-CPPs are shown in blue. **Fig F.** UMAP visualization of the *mlcpp* independent test dataset for the CPP Classification task. The UMAP model was fitted on the CPP2Vec-GenSet training dataset, and the test embeddings were projected onto the same embedding space. CPPs are depicted in orange, while Non-CPPs are shown in blue. **Fig G.** UMAP visualization of a representative validation fold for the CPP Classification task. The UMAP model was fitted on CPP2Vec-GenSet, and the validation embeddings were projected onto the same embedding space. CPPs are depicted in orange, while Non-CPPs are shown in blue. **Fig H.** UMAP visualization of CPPsite3_Gautam Dataset for the Uptake Efficiency task. The UMAP model was fitted on this training dataset and the embeddings of the validation and independent test sequences were projected onto the same embedding space. Peptides with high uptake efficiency are depicted in orange, while those with low uptake efficiency are shown in blue. **Fig I.** UMAP visualization of the 64-peptides independent test dataset for the Uptake Efficiency task. The UMAP model was fitted on the CPPsite3_Gautam training dataset, and the test embeddings were projected onto the same embedding space. Peptides with high uptake efficiency are depicted in orange, while those with low uptake efficiency are shown in blue. **Fig J.** UMAP visualization of a representative validation fold for the Uptake Efficiency task. The UMAP model was fitted on CPPsite3_Gautam dataset, and the validation embeddings were projected onto the same embedding space. Peptides with high uptake efficiency are depicted in orange, while those with low uptake efficiency are shown in blue. **Fig K.** UMAP visualization of the 64-peptides dataset for the PMO Delivery task. The UMAP model was fitted on this training dataset, and the embeddings of the validation folds and the independent 7-novel-sequences dataset were projected onto the same embedding space. Peptides exhibiting ≥3-fold improvement in eGFP fluorescence relative to unconjugated PMO are depicted in orange, whereas peptides with <3-fold improvement are shown in blue. **Fig L.** UMAP visualization of the independent 7-novel-sequences dataset for the PMO Delivery task. The UMAP model was fitted on the 64-peptides training dataset, and the embeddings of the novel sequences were projected onto the same embedding space. Peptides exhibiting ≥3-fold improvement in eGFP fluorescence relative to unconjugated PMO are depicted in orange, whereas peptides with <3-fold improvement are shown in blue. **Fig M.** UMAP visualization of a representative 3-fold validation split for the PMO Delivery task. The UMAP model was fitted on the 64-peptides training dataset, and the embeddings of the validation peptides were projected onto the same embedding space. Peptides exhibiting ≥3-fold improvement in eGFP fluorescence relative to unconjugated PMO are depicted in orange, whereas peptides with <3-fold improvement are shown in blue. **Table F.** Cluster statistics of W2V embeddings for the Uptake Efficiency task across training, validation, and independent test sets. **Table G.** Cluster statistics of W2V embeddings for the PMO Delivery task across training, validation, and independent test sets. **Fig N.** Heatmap of the first five PCA Components for the *kelm* dataset. Peptides are sorted by labels from CPPs to Non-CPPs and by descending predicted probability score. The black vertical line illustrates the boundary between classes. **Fig O.** Heatmap of the first five PCA Components for the 64-peptides dataset. Peptides are sorted by labels from High to Low Uptake Efficiency and by descending predicted probability score. The black vertical line illustrates the boundary between classes. **Fig P.** Heatmap of the first five PCA Components for the 7-novel-sequences dataset. Peptides are sorted by labels from ≥ 3-fold to <3-fold improvement in eGFP fluorescence compared to unconjugated PMOs and by descending predicted probability score. The black vertical line illustrates the boundary between classes. **Table H.** Performance of CPP2Vec and CPP2LLM compared to 12 CPP prediction models on *kelm* dataset. **Table I.** Performance of CPP2Vec compared to 12 CPP prediction models on *mlcpp* dataset.
(PDF)

S2 Text Selected results of parameter optimization experiments for Word2Vec embeddings and machine learning classifiers. (XLSX)
