# Supplementary material for: CPP2Vec: A representation learning approach for cell-penetrating peptides prediction
Source: PLoS Comput Biol. 2026 Jul 13;22(7):e1014118. doi: 10.1371/journal.pcbi.1014118 (PMC13411874; doi:10.1371/journal.pcbi.1014118)
Supplement: S1 Text — Table A. Word2Vec hyperparameter tuning for CPP2Vec. Table B. Hyperparameters of the pre-trained CPP2LLM models. Table C. Hyperparameter search regions per machine learning or deep learning method. Table D. The average metrics for StratifiedKFold. Table E. The average metrics for KFold. Fig A. UMAP visualization of mlcpp dataset. With orange are represented the CPPs while with blue the Non-CPPs. Fig B. UMAP visualization of 64-peptides dataset. With orange are noted the peptides with high uptake efficiency, while with blue those with low uptake efficiency. Fig C. UMAP visualization of 7-novel-sequences dataset. With orange are depicted the peptides with a ≥ 3-fold improvement in eGFP fluorescence compared to unconjugated PMOs, while with blue those with <3-fold improvement. Fig D. UMAP visualization of CPP2Vec-GenSet for the CPP Classification task. The UMAP model was fitted on this training dataset (i.e., CPP2Vec-GenSet) and the embeddings of the validation and independent test sequences were projected onto the same embedding space. CPPs are depicted in orange, while Non-CPPs are shown in blue. Fig E. UMAP visualization of the kelm independent test dataset for the CPP Classification task. The UMAP model was fitted on the CPP2Vec-GenSet training dataset, and the test embeddings were projected onto the same embedding space. CPPs are depicted in orange, while Non-CPPs are shown in blue. Fig F. UMAP visualization of the mlcpp independent test dataset for the CPP Classification task. The UMAP model was fitted on the CPP2Vec-GenSet training dataset, and the test embeddings were projected onto the same embedding space. CPPs are depicted in orange, while Non-CPPs are shown in blue. Fig G. UMAP visualization of a representative validation fold for the CPP Classification task. The UMAP model was fitted on CPP2Vec-GenSet, and the validation embeddings were projected onto the same embedding space. CPPs are depicted in orange, while Non-CPPs are shown in blue. Fig H. UMAP visua [file pcbi.1014118.s001.pdf]

CPP2Vec: a Representation Learning Approach for Cell-Penetrating Peptides Prediction

Stavroula Svolou, Vasileios Konstantakos, Anastasia Krithara and Georgios Paliouras

Supplementary Material

Table A in S1 Text. Word2Vec hyperparameter tuning for CPP2Vec.

Representation Approach	Hyperparameter	Search Region
Word2Vec	seqwin	[30, 35, 40, 45, 50, 55, 60]
	vector_size	[100, 200, 300, 400]
	epochs	[4 - 20]
	skip_gram	[0, 1]
	window_size	[5, 10, 15, 20, 25, 30, 35, 40]

Table B in S1 Text. Hyperparameters of the pre-trained CPP2LLM models.

Representation Approach	Hyperparameter	Value
T5	Pre-training dataset	UniRef50
	Number of parameters	3B
	Hidden size	1024
	Number of layers	24
	Number of attention heads	32
	Dimension of feedforward layer	4096
ProtT5-XL-UniRef50	Sequence length	seqwin
BERT	Pre-training dataset	UniRef100 & Swiss-Prot
	Number of parameters	420M
	Hidden size	1024
	Number of layers	24
	Number of attention heads	16
	Dimension of feedforward layer	4096
ProtBERT	Sequence length	seqwin
ESM-2	Pre-training dataset	UniRef50
	Number of parameters	3B
	Hidden size	2560
	Number of layers	36
	Number of attention heads	40
	Dimension of feedforward layer	10240
esm2_t36_3B_UR50D	Sequence length	seqwin

seqwin: 36 (CPP-Classification), 61 (Uptake-Efficiency), and 27 (PMO-Delivery).

Table C in S1 Text. Hyperparameter search regions per machine learning or deep learning method.

ML Model	Hyperparameter	Search Region
RF	max_features	[0.25, 0.5, 0.75]
	n_estimators	[10, 50, 250, 1000]
	max_depth	[2, 4, 5, 6, 7, 8, 10, 20]
SVM	C	[1.0, 5.0, 10.0]
	Gamma	[0.1, 0.01, 0.001, 'auto', 'scale']
	Kernel	['linear', 'poly', 'rbf']
	Probability	['True', 'False']
GB	n_estimators	100
	learning_rate	[0.1, 1.0]
	max_depth	[1, 4]
TX	n_layers	[2, 3, 4]
	n_heads	[4, 5, 6, 7, 8]
	d_dim	[100, 128]
	d_ff	[256, 400]
CNN	features	vector_size
	time_size	seqwin-kmer+1
	batch_size	128
bLSTM	hidden_size	128
	n_layers	[1, 2, 3]
	learning_rate	[0.001, 0.1]
	batch_size	[64, 128]

For the CPP-Classification, Uptake-Efficiency and PMO-Delivery tasks (seqwin, kmer, vector_size) parameters are: (36, 2, 200), (61, 3, 300), and (27, 1, 100), respectively.

Table D in S1 Text. The average metrics for StratifiedKFold.

Dataset	Accuracy	MCC	AUC	Precision	Recall
**Model = RF (max_features=0.75, max_depth=20, random_state=0, n_estimators=50)**					
Validation	0.713	0.421	0.803	0.667	0.679
Test	0.794	0.682	0.961	1.000	0.711
**Model = RF (max_features=0.5, max_depth=20, random_state=0, n_estimators=250)**					
Validation	0.734	0.463	0.791	0.692	0.691
Test	0.714	0.602	0.961	1.000	0.600

MCC: Matthews Correlation Coefficient, AUC: Area Under Curve.

Table E in S1 Text. The average metrics for KFold.

Dataset	Accuracy	MCC	AUC	Precision	Recall
**Model = RF (max_features=0.75, max_depth=20, random_state=0, n_estimators=50)**					
Validation	0.741	0.489	0.828	0.714	0.694
Test	0.730	0.565	0.928	0.963	0.644
**Model = RF (max_features=0.5, max_depth=20, random_state=0, n_estimators=250)**					
Validation	0.761	0.506	0.842	0.753	0.635
Test	0.778	0.652	0.994	1.000	0.689

MCC: Matthews Correlation Coefficient, AUC: Area Under Curve.

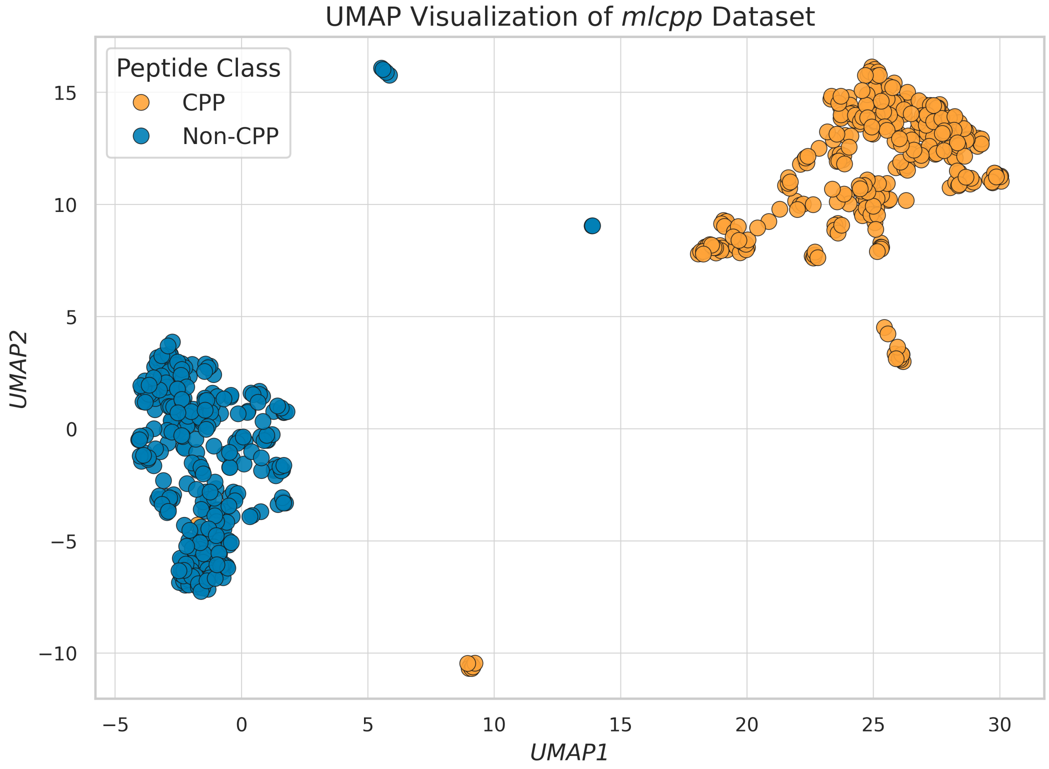

Fig A in S1 Text. UMAP visualization of *mlcpp* dataset. With orange are represented the CPPs while with blue the Non-CPPs.

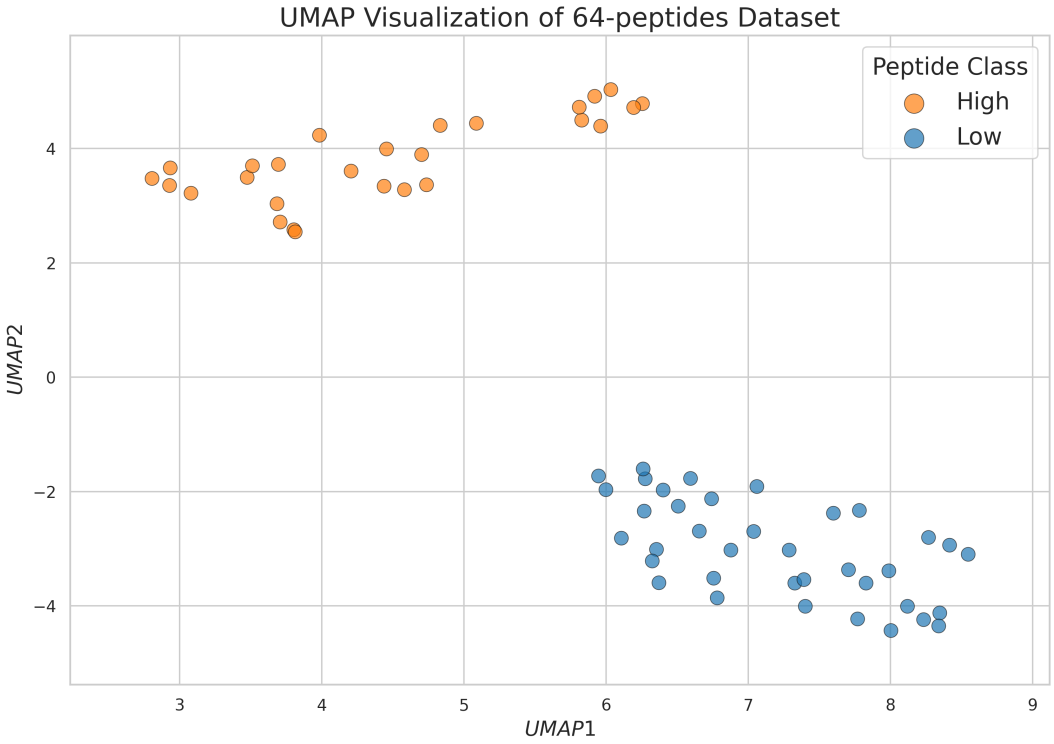

Fig B in S1 Text. UMAP visualization of 64-peptides dataset. With orange are noted the peptides with high uptake efficiency, while with blue those with low uptake efficiency.

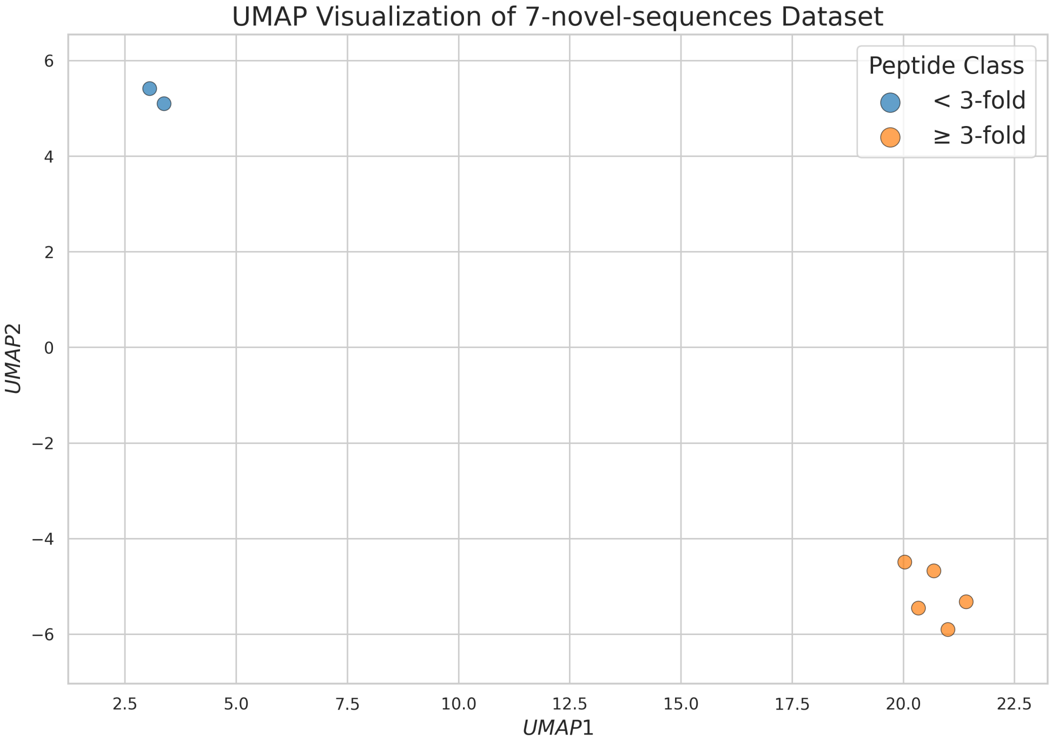

Fig C in S1 Text. UMAP visualization of 7-novel-sequences dataset. With orange are depicted the peptides with a $\geq$ 3-fold improvement in eGFP fluorescence compared to unconjugated PMOs, while with blue those with <3-fold improvement.

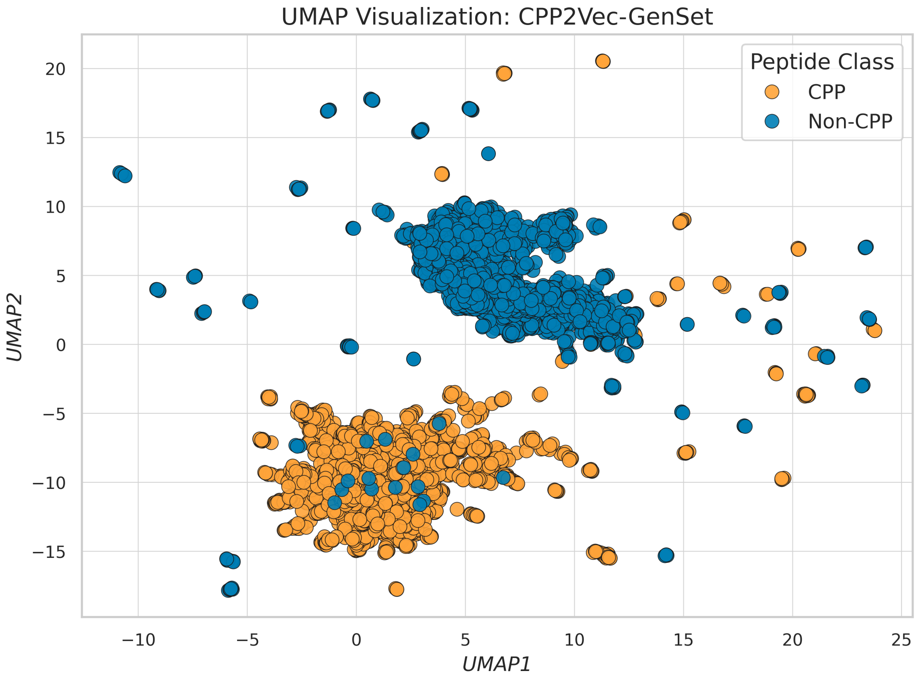

Fig D in S1 Text. UMAP visualization of CPP2Vec-GenSet for the CPP Classification task. The UMAP model was fitted on this training dataset (i.e., CPP2Vec-GenSet) and the embeddings of the validation and independent test sequences were projected onto the same embedding space. CPPs are depicted in orange, while Non-CPPs are shown in blue.

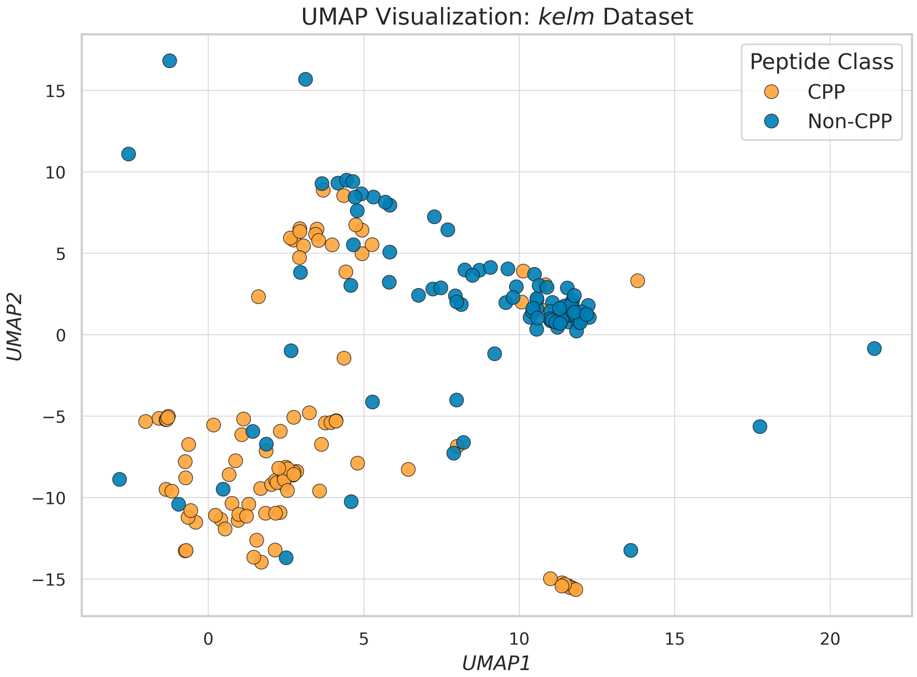

Fig E in S1 Text. UMAP visualization of the *kelm* independent test dataset for the CPP Classification task. The UMAP model was fitted on the CPP2Vec-GenSet training dataset, and the test embeddings were projected onto the same embedding space. CPPs are depicted in orange, while Non-CPPs are shown in blue.

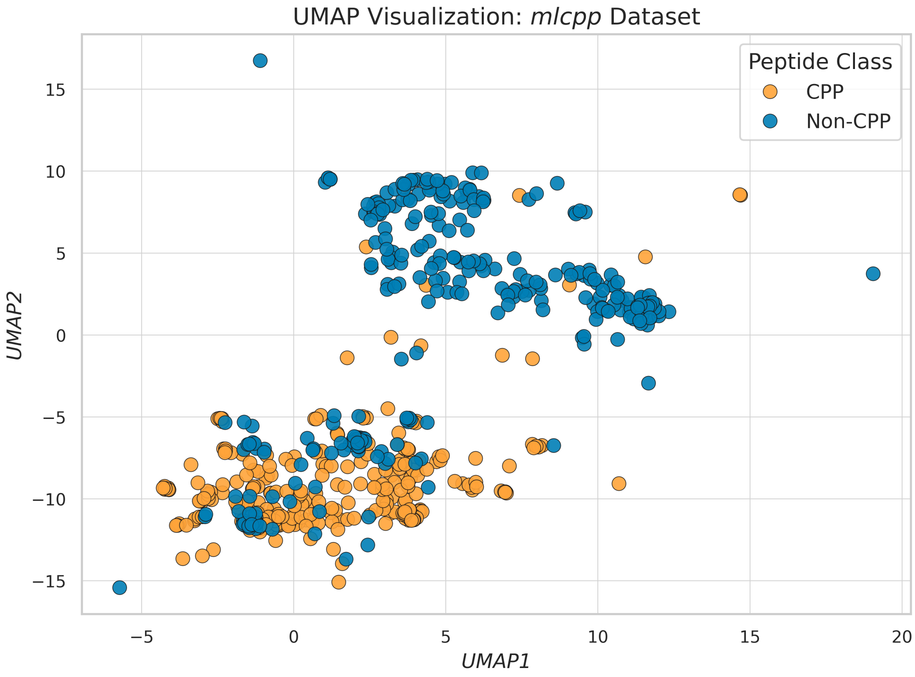

Fig F in S1 Text. UMAP visualization of the *mlcpp* independent test dataset for the CPP Classification task. The UMAP model was fitted on the CPP2Vec-GenSet training dataset, and the test embeddings were projected onto the same embedding space. CPPs are depicted in orange, while Non-CPPs are shown in blue.

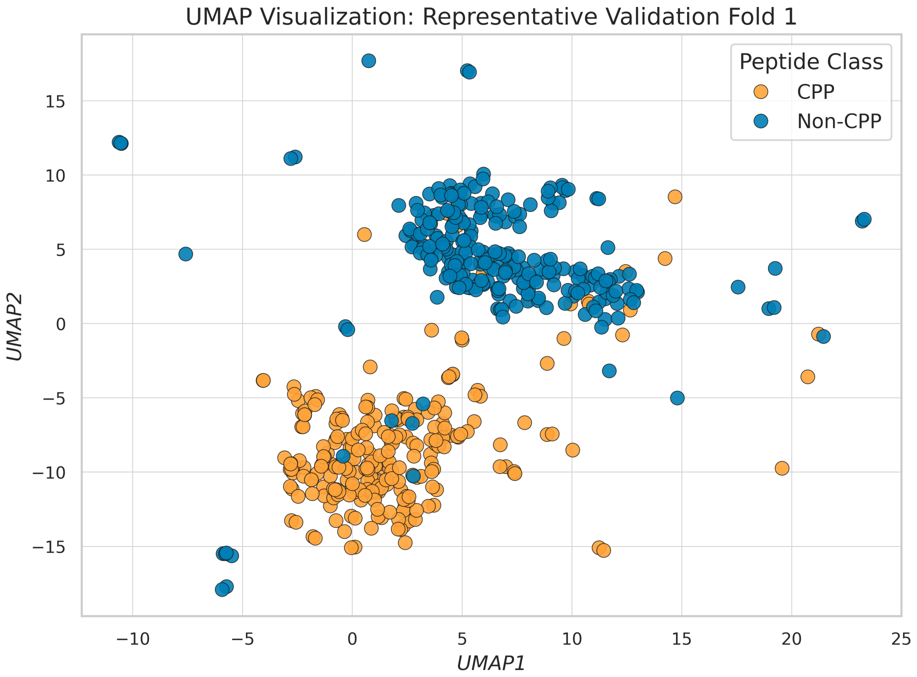

Fig G in S1 Text. UMAP visualization of a representative validation fold for the CPP Classification task. The UMAP model was fitted on CPP2Vec-GenSet, and the validation embeddings were projected onto the same embedding space. CPPs are depicted in orange, while Non-CPPs are shown in blue.

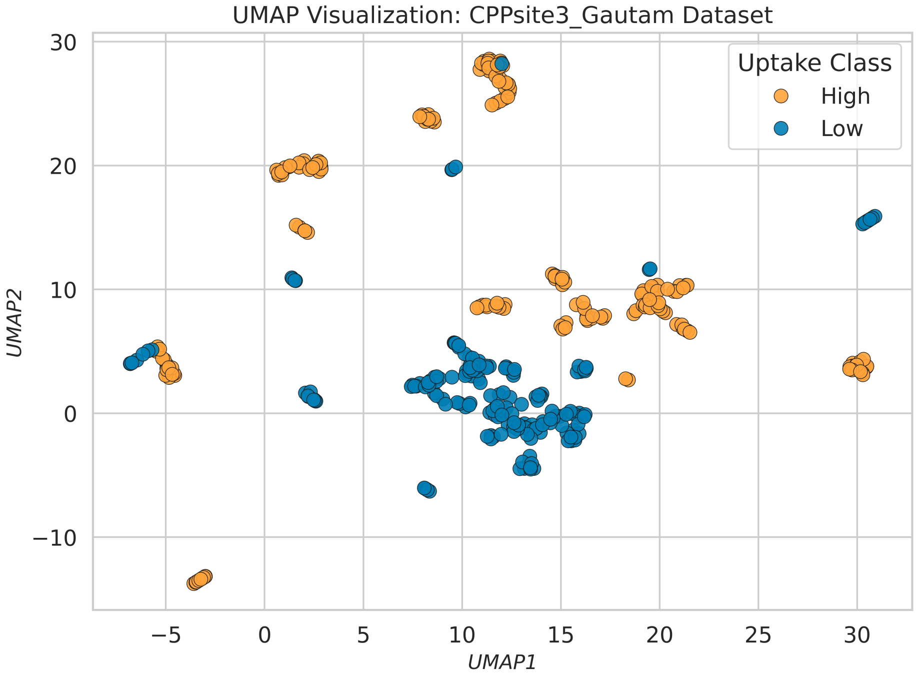

Fig H in S1 Text. UMAP visualization of CPPsite3_Gautam Dataset for the Uptake Efficiency task. The UMAP model was fitted on this training dataset and the embeddings of the validation and independent test sequences were projected onto the same embedding space. Peptides with high uptake efficiency are depicted in orange, while those with low uptake efficiency are shown in blue.

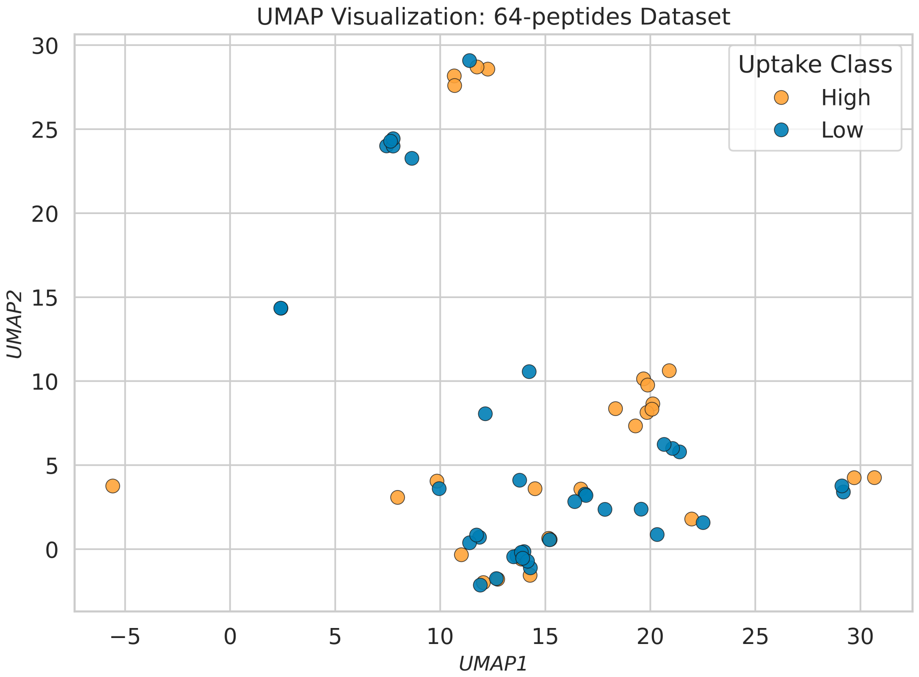

Fig I in S1 Text. UMAP visualization of the 64-peptides independent test dataset for the Uptake Efficiency task. The UMAP model was fitted on the CPPsite3_Gautam training dataset, and the test embeddings were projected onto the same embedding space. Peptides with high uptake efficiency are depicted in orange, while those with low uptake efficiency are shown in blue.

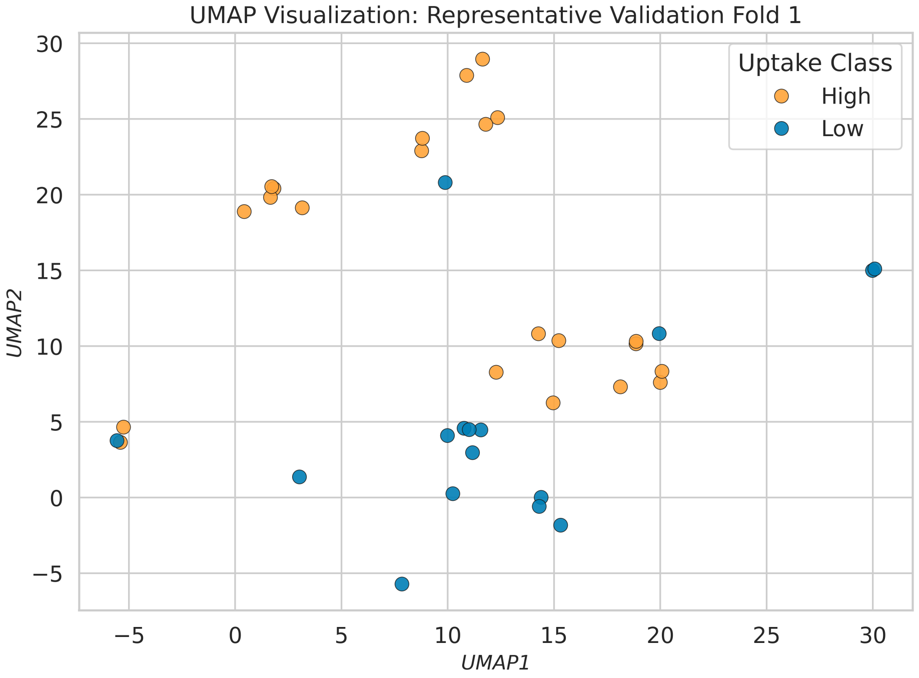

Fig J in S1 Text. UMAP visualization of a representative validation fold for the Uptake Efficiency task. The UMAP model was fitted on CPPsite3_Gautam dataset, and the validation embeddings were projected onto the same embedding space. Peptides with high uptake efficiency are depicted in orange, while those with low uptake efficiency are shown in blue.

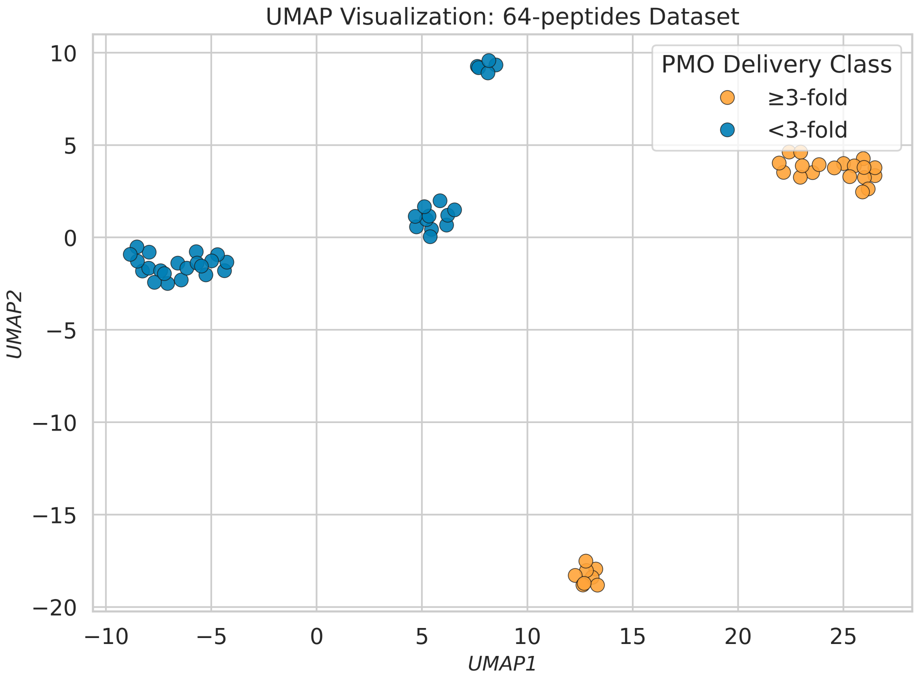

Fig K in S1 Text. UMAP visualization of the 64-peptides dataset for the PMO Delivery task. The UMAP model was fitted on this training dataset, and the embeddings of the validation folds and the independent 7-novel-sequences dataset were projected onto the same embedding space. Peptides exhibiting $\geq$3-fold improvement in eGFP fluorescence relative to unconjugated PMO are depicted in orange, whereas peptides with $<$3-fold improvement are shown in blue.

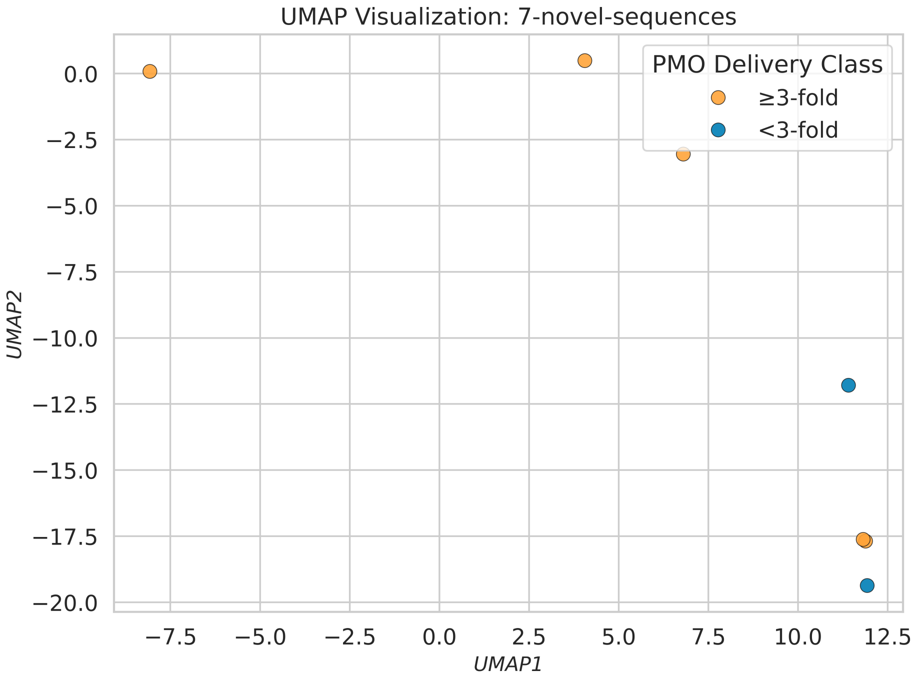

Fig L in S1 Text. UMAP visualization of the independent 7-novel-sequences dataset for the PMO Delivery task. The UMAP model was fitted on the 64-peptides training dataset, and the embeddings of the novel sequences were projected onto the same embedding space. Peptides exhibiting $\geq$3-fold improvement in eGFP fluorescence relative to unconjugated PMO are depicted in orange, whereas peptides with $<$3-fold improvement are shown in blue.

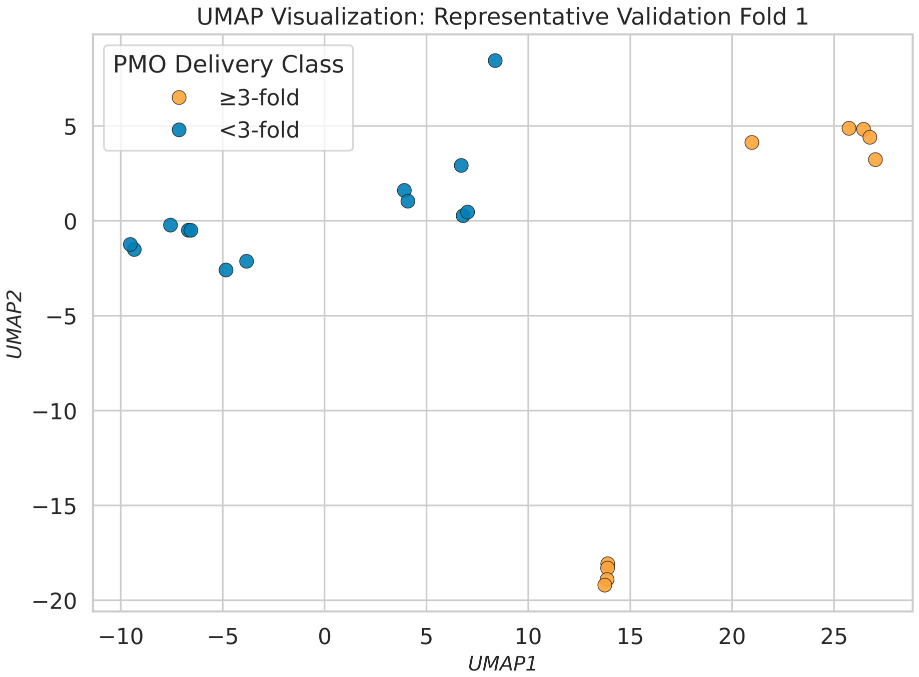

Fig M in S1 Text. UMAP visualization of a representative 3-fold validation split for the PMO Delivery task. The UMAP model was fitted on the 64-peptides training dataset, and the embeddings of the validation peptides were projected onto the same embedding space. Peptides exhibiting ≥3-fold improvement in eGFP fluorescence relative to unconjugated PMO are depicted in orange, whereas peptides with <3-fold improvement are shown in blue.

Table F in S1 Text. Cluster statistics of W2V embeddings for the Uptake Efficiency task across training, validation, and independent test sets.

Dataset	Silhouette Score	Mean Intra-class Distance	Mean Inter-class Distance	Inter/Intra Ratio
**CPPsite3_Gautam (Training)**	0.2522	13.1441	17.8514	1.3581
**Validation (10-fold mean)**	0.2218	13.4328	17.4496	1.3095
**64-peptides (Test)**	0.0137	13.0168	13.2356	1.0168

Cluster statistics were computed from the W2V embeddings projected via UMAP fitted on the CPPsite3_Gautam training dataset for the Uptake Efficiency task. Silhouette score measures cluster separation; mean intra-class and inter-class distances quantify within-class compactness and between-class separation; the inter/intra ratio indicates relative class separation. Validation statistics are averaged across 10-fold stratified cross-validation splits. Mann–Whitney U test p-values were < 0.001 for both the training and validation datasets, and $p = 0.1915$ for the 64-peptides independent test set.

Table G in S1 Text. Cluster statistics of W2V embeddings for the PMO Delivery task across training, validation, and independent test sets.

Dataset	Silhouette Score	Mean Intra-class Distance	Mean Inter-class Distance	Inter/Intra Ratio
**64-peptides (Training)**	0.5932	9.7373	25.5790	2.6269
**Validation (3-fold mean)**	0.5709	10.3701	25.8475	2.5020
**7-novel-sequences (Test)**	-0.0313	14.8376	12.8531	0.8663

Cluster statistics were computed from the W2V embeddings projected via UMAP fitted on the 64-peptides training dataset for the PMO Delivery task. Silhouette score measures cluster separation; mean intra-class and inter-class distances quantify within-class compactness and between-class separation; the inter/intra ratio indicates relative class separation. Validation statistics are averaged across 3-fold stratified cross-validation splits. Mann–Whitney U test p-values were < 0.0001 for the training and validation datasets and 0.676 for the 7-novel-sequences independent test set.

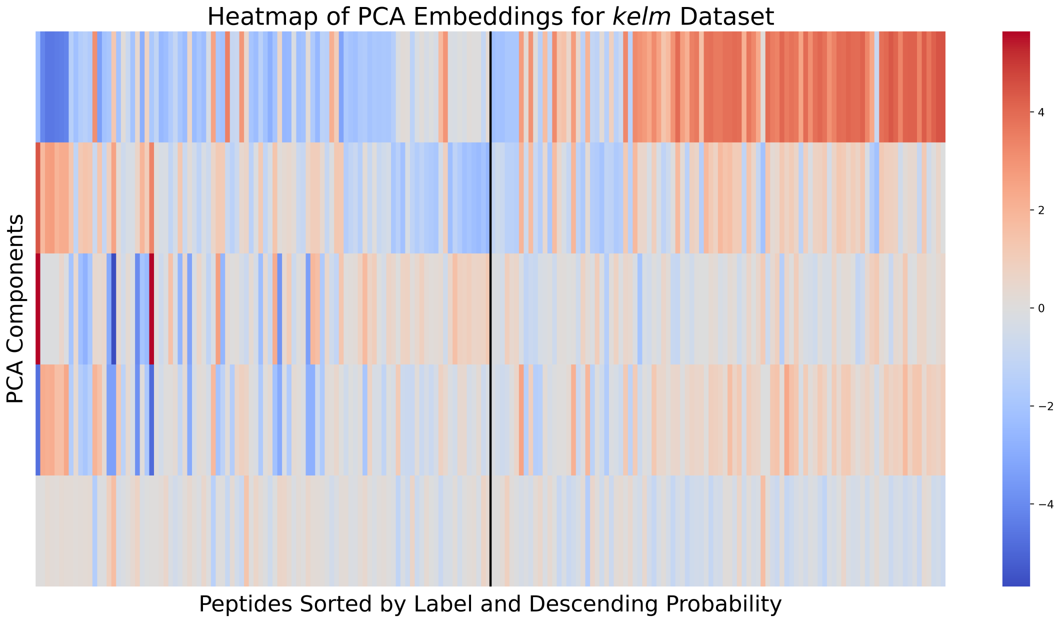

Fig N in S1 Text. Heatmap of the first five PCA Components for the *kelm* dataset. Peptides are sorted by labels from CPPs to Non-CPPs and by descending predicted probability score. The black vertical line illustrates the boundary between classes.

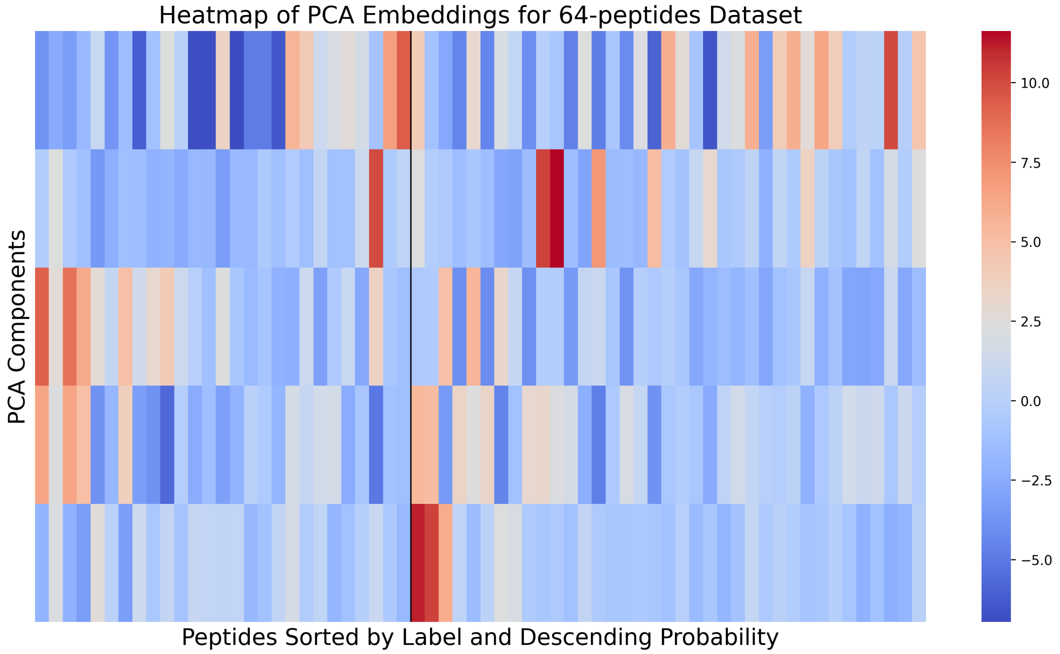

Fig O in S1 Text. Heatmap of the first five PCA Components for the 64-peptides dataset. Peptides are sorted by labels from High to Low Uptake Efficiency and by descending predicted probability score. The black vertical line illustrates the boundary between classes.

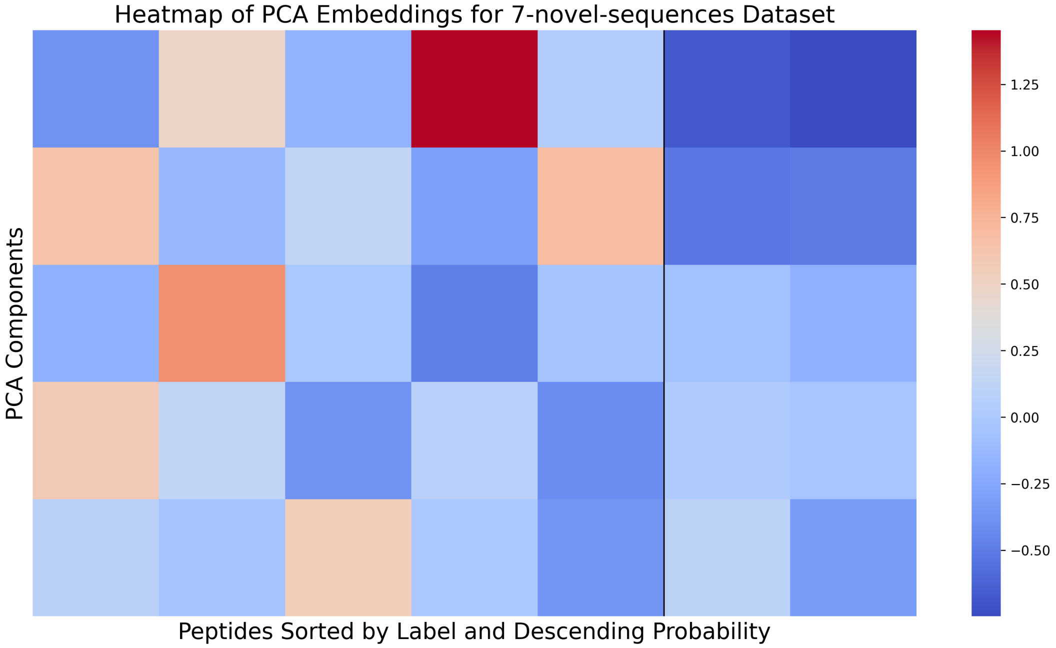

Fig P in S1 Text. Heatmap of the first five PCA Components for the 7-novel-sequences dataset. Peptides are sorted by labels from ≥ 3-fold to <3-fold improvement in eGFP fluorescence compared to unconjugated PMOs and by descending predicted probability score. The black vertical line illustrates the boundary between classes.

Table H in S1 Text. Performance of CPP2Vec and CPP2LLM compared to 12 CPP prediction models on *kelm* dataset.

Prediction Tool	Sensitivity (%)	Specificity (%)	Accuracy (%)	MCC
pLM4CPPs (ESM-480)	80.20	92.70	86.50	0.74
MLCPP 2.0	74.00	96.90	85.40	0.73
MLCPP	74.65	89.58	80.67	0.63
CPPred-RF	**83.10**	75.00	79.83	0.58
KELM-AAC	69.01	89.58	77.31	0.58
KELM-hybrid-AAC	69.01	89.58	77.31	0.58
CPPred-FL	78.87	79.17	78.99	0.57
CellPPD	63.38	93.75	75.63	0.57
CellPPD-motif	63.38	93.75	75.63	0.57
KELM-PseAAC	**83.10**	72.92	78.99	0.56
KELM-DAC	56.34	**97.92**	73.11	0.56
SkipCPP-Pred	81.69	72.92	78.15	0.55
KELM-hybrid-PseAAC	**83.10**	70.83	78.15	0.54
KELM-hybrid-DAC	69.01	83.33	74.79	0.51
CPP2Vec	79.90	94.10	**87.00**	**0.75**
CPP2LLM	79.50	88.40	84.00	0.68

MCC: Matthews Correlation Coefficient.

Table I in S1 Text. Performance of CPP2Vec compared to 12 CPP prediction models on *mlcpp* dataset.

Prediction Tool	Sensitivity (%)	Specificity (%)	Accuracy (%)	MCC
pLM4CPPs (ESM-480)	95.18	95.18	95.18	0.90
KELM-hybrid-AAC	94.63	**96.37**	**95.61**	**0.91**
KELM-hybrid-DAC	94.63	73.06	82.46	0.68
KELM-AAC	93.96	73.06	82.16	0.67
KELM-PseAAC	95.30	70.47	81.29	0.66
CPPred-FL	96.64	67.88	80.41	0.65
MLCPP	96.64	66.32	79.53	0.64
CPPred-RF	97.99	61.66	77.49	0.62
SkipCPP-Pred	**99.33**	58.03	76.02	0.60
CellPPD	80.54	75.65	77.78	0.56
CellPPD-motif	80.54	75.65	77.78	0.56
KELM-hybrid-PseAAC	92.62	56.48	72.22	0.51
KELM-DAC	92.62	54.92	71.35	0.50
CPP2Vec	98.80	75.50	87.10	0.76
CPP2LLM	98.30	69.50	83.90	0.71

MCC: Matthews Correlation Coefficient.
